# Association between the 2008–09 Seasonal Influenza Vaccine and Pandemic H1N1 Illness during Spring–Summer 2009: Four Observational Studies from Canada

**DOI:** 10.1371/journal.pmed.1000258

**Published:** 2010-04-06

**Authors:** Danuta M. Skowronski, Gaston De Serres, Natasha S. Crowcroft, Naveed Z. Janjua, Nicole Boulianne, Travis S. Hottes, Laura C. Rosella, James A. Dickinson, Rodica Gilca, Pam Sethi, Najwa Ouhoummane, Donald J. Willison, Isabelle Rouleau, Martin Petric, Kevin Fonseca, Steven J. Drews, Anuradha Rebbapragada, Hugues Charest, Marie-Ève Hamelin, Guy Boivin, Jennifer L. Gardy, Yan Li, Trijntje L. Kwindt, David M. Patrick, Robert C. Brunham, for the Canadian SAVOIR Team

**Affiliations:** 1 British Columbia Centre for Disease Control (BCCDC), Vancouver, British Columbia, Canada; 2 Institut national de santé publique du Québec, Québec, Canada; 3 Ontario Agency for Health Protection and Promotion, Toronto, Ontario, Canada; 4 Dalla Lana School of Public Health, University of Toronto, Toronto, Ontario, Canada; 5 University of Calgary, Calgary, Alberta, Canada; 6 Alberta Provincial Laboratory, Alberta, Canada; 7 Centre Hospitalier Universitaire de Québec (CHUQ) Research Center, Laval University, Québec, Canada; 8 National Microbiology Laboratory, Public Health Agency of Canada, Winnipeg, Manitoba, Canada; George Washington University, United States of America

## Abstract

**Background:**

In late spring 2009, concern was raised in Canada that prior vaccination with the 2008–09 trivalent inactivated influenza vaccine (TIV) was associated with increased risk of pandemic influenza A (H1N1) (pH1N1) illness. Several epidemiologic investigations were conducted through the summer to assess this putative association.

**Methods and Findings:**

Studies included: (1) test-negative case-control design based on Canada's sentinel vaccine effectiveness monitoring system in British Columbia, Alberta, Ontario, and Quebec; (2) conventional case-control design using population controls in Quebec; (3) test-negative case-control design in Ontario; and (4) prospective household transmission (cohort) study in Quebec. Logistic regression was used to estimate odds ratios for TIV effect on community- or hospital-based laboratory-confirmed seasonal or pH1N1 influenza cases compared to controls with restriction, stratification, and adjustment for covariates including combinations of age, sex, comorbidity, timeliness of medical visit, prior physician visits, and/or health care worker (HCW) status. For the prospective study risk ratios were computed. Based on the sentinel study of 672 cases and 857 controls, 2008–09 TIV was associated with statistically significant protection against seasonal influenza (odds ratio 0.44, 95% CI 0.33–0.59). In contrast, estimates from the sentinel and three other observational studies, involving a total of 1,226 laboratory-confirmed pH1N1 cases and 1,505 controls, indicated that prior receipt of 2008–09 TIV was associated with increased risk of medically attended pH1N1 illness during the spring–summer 2009, with estimated risk or odds ratios ranging from 1.4 to 2.5. Risk of pH1N1 hospitalization was not further increased among vaccinated people when comparing hospitalized to community cases.

**Conclusions:**

Prior receipt of 2008–09 TIV was associated with increased risk of medically attended pH1N1 illness during the spring–summer 2009 in Canada. The occurrence of bias (selection, information) or confounding cannot be ruled out. Further experimental and epidemiological assessment is warranted. Possible biological mechanisms and immunoepidemiologic implications are considered.

*Please see later in the article for the Editors' Summary*

## Abstract 2

In three case-control studies and a household transmission cohort, Danuta Skowronski and colleagues find an association between prior seasonal flu vaccination and increased risk of 2009 pandemic H1N1 flu.

## Editors' Summary

**Background:**

Every winter, millions of people catch influenza—a viral infection of the airways—and hundreds of thousands of people die as a result. These seasonal epidemics occur because small but frequent changes in the influenza virus mean that an immune response produced one year through infection or vaccination provides only partial protection against influenza the next year. Annual vaccination with killed influenza viruses of the major circulating strains can greatly reduce a person's risk of catching influenza. Consequently, many countries run seasonal influenza vaccination programs. In most of Canada, vaccination with a mixture of three inactivated viruses (a trivalent inactivated vaccine or TIV) is provided free to children aged 6–23 months, to elderly people, to people with long-term conditions that increase their risk of influenza-related complications, and those who provide care for them; in Ontario, free vaccination is offered to everyone older than 6 months.

In addition, influenza viruses occasionally emerge that are very different and to which human populations have virtually no immunity. These viruses can start global epidemics (pandemics) that can kill millions of people. Experts have been warning for some time that an influenza pandemic is long overdue and, in March 2009, the first cases of influenza caused by a new virus called pandemic A/H1N1 2009 (pH1N1; swine flu) occurred in Mexico. The virus spread rapidly and on 11 June 2009, the World Health Organization declared that a global pandemic of pH1N1 influenza was underway. By the end of February 2010, more than 16,000 people around the world had died from pH1N1.

**Why Was This Study Done?:**

During an investigation of a school outbreak of pH1N1 in the late spring 2009 in Canada, investigators noted that people with illness characterized by fever and coughing had been vaccinated against seasonal influenza more often than individuals without such illness. To assess whether this association between prior vaccination with seasonal 2008–09 TIV and subsequent pH1N1 illness was evident in other settings, researchers in Canada therefore conducted additional studies using different methods. In this paper, the researchers report the results of four additional studies conducted in Canada during the summer of 2009 to assess this possible association.

**What Did the Researchers Do and Find?:**

The researchers conducted four epidemiologic studies. Epidemiology is the study of the causes, distribution, and control of diseases in populations.

Three of the four studies were case-control studies in which the researchers assessed the frequency of prior vaccination with the 2008–09 TIV in people with pH1N1 influenza compared to the frequency among healthy members of the general population or among individuals who had an influenza-like illness but no sign of infection with an influenza virus. The researchers also did a household transmission study in which they collected information about vaccination with TIV among the additional cases of influenza that were identified in 47 households in which a case of laboratory-confirmed pH1N1 influenza had occurred. The first of the case-control studies, which was based on Canada's vaccine effectiveness monitoring system, showed that, as expected, the 2008–09 TIV provided protection against seasonal influenza. However, estimates from all four studies (which included about 1,200 laboratory-confirmed pH1N1 cases and 1,500 controls) showed that prior recipients of the 2008–09 TIV had approximately 1.4–2.5 times increased chances of developing pH1N1 illness that needed medical attention during the spring–summer of 2009 compared to people who had not received the TIV. Prior seasonal vaccination was not associated with an increase in the severity of pH1N1 illness, however. That is, it did not increase the risk of being hospitalized among those with pH1N1 illness.

**What Do These Findings Mean?:**

Because all the investigations in this study are “observational,” the people who had been vaccinated might share another unknown characteristic that is actually responsible for increasing their risk of developing pH1N1 illness (“confounding”). Furthermore, the results reported in this study might have arisen by chance, although the consistency of results across the studies makes this unlikely. Thus, the finding of an association between prior receipt of 2008–09 TIV and an increased risk of pH1N1 illness is not conclusive and needs to be investigated further, particularly since some other observational studies conducted in other countries have reported that seasonal vaccination had no influence or may have been associated with reduced chances of pH1N1 illness. If the findings in the current study are real, however, they raise important questions about the biological interactions between seasonal and pandemic influenza strains and vaccines, and about the best way to prevent and control both types of influenza in future.

**Additional Information:**

Please access these Web sites via the online version of this summary at http://dx.doi.org/ 10.1371/journal.pmed.1000258.

- This article is further discussed in a *PLoS Medicine* Perspective by Cécile Viboud and Lone Simonsen
- FightFlu.ca, a Canadian government Web site, provides access to information on pH1N1 influenza
- The US Centers for Disease Control and Prevention provides information about influenza for patients and professionals, including specific information on H1N1 influenza
- Flu.gov, a US government website, provides access to information on H1N1, avian and pandemic influenza
- The World Health Organization provides information on seasonal influenza and has detailed information on pH1N1 influenza (in several languages)
- The UK Health Protection Agency provides information on pandemic influenza and on pH1N1 influenza

## Introduction

On 17 April 2009 a novel swine-origin influenza A (H1N1) virus was identified as the cause of two pediatric cases of febrile respiratory illness in California [1],[2]. Shortly thereafter, this virus was also identified as the cause of an outbreak of severe respiratory illness occurring among young people in Mexico in March and April [3],[4]. Subsequent spread throughout North America and elsewhere resulted in the declaration, on 11 June, of a phase 6 pandemic of the novel influenza A (H1N1) (now called pandemic influenza A (H1N1) [pH1N1]) by the World Health Organization (WHO) [5]. Early surveillance and immunogenicity data and global summary by the WHO emphasized increased risk among young people <50 years of age [2],[6],[7].

Influenza vaccine is recommended and provided free in Canada to children 6–23 months of age, elderly people, people of all ages with designated high-risk conditions, and their household contacts and care providers [8]. In Ontario, all residents ≥6 months of age can access the influenza vaccine annually free of charge via a universal immunization program initiated in 2000. Residents of other provinces who are not among the high-risk or their contacts are also encouraged to receive influenza vaccine, and this may be provided free by their employers or they must purchase it. In its annual update, WHO recommended changes to all three components of the influenza vaccine for the 2008–09 season [8]. GlaxoSmithKline (GSK Fluviral; domestically manufactured) and Sanofi Pasteur (Vaxigrip; imported) supply approximately 75% and 25%, respectively, of the split trivalent inactivated influenza vaccine (TIV) distributed each year in Canada; Quebec generally receives a higher proportion of GlaxoSmithKline product (∼95% for 2008–09 and 2007–08) while Ontario generally receives less (∼67% for 2008–09 and 2007–08). Live attenuated influenza vaccine is not yet approved for use in Canada.

During the last week of April, a school pH1N1 outbreak was reported in a rural community of northern British Columbia (BC) [9]. During outbreak investigation, an unexpected signal of association between prior vaccination with TIV and fever/cough illness was noted. Because these unexpected results were based on a nonspecific outcome defined by clinical syndrome, separate follow-up studies were arranged to investigate this initial signal using more stringent methodologies based on laboratory-confirmed outcomes. We report the results of four epidemiologic studies conducted during the summer of 2009 in Canada to assess the putative association between vaccination with seasonal 2008–09 TIV and pH1N1 illness.

## Methods

The design, sample size, and outcomes evaluated in each of the four studies to assess the association between prior 2008–09 TIV receipt and risk of pH1N1 illness are summarized in Table 1.

**Table 1:** Summary of study design, sample size and outcomes applied in evaluating the association between 2008–09 TIV receipt and pH1N1 risk.

Study and Design	Study Location	Study Period	Cases	Controls	Number of Cases	Number of Controls	Effect Measure	Covariates	Secondary Analyses
**Sentinel test-negative case-control**	Sentinel sites in British Columbia, Alberta, Ontario, Quebec	ILI onset spanning:**Seasonal:**1 November 2008 to 31 March 2009**pH1N1:**17 April to 22 July 2009	Medically attended ILI test-positive for seasonal or pH1N1 influenza	Medically attended ILI test-negative for seasonal and pH1N1 influenza	**Seasonal:** 672**pH1N1:**144	**Seasonal:**857**pH1N1:**536	OR	Age, comorbidity, province, interval between ILI onset and specimen collection	2007-08 TIV receipt; age stratified (50 y). Restriction to the period after May 17, to young adults, to provinces of Quebec and Ontario and to those without comorbidity also explored.
**Quebec population case-control**	The four regions of Quebec with >75% of community and hospitalized pH1N1 cases	Household telephone survey of cases and controls conducted17 July to 10 August 2009	Medically attended pH1N1 test-positive non-hospitalized and hospitalized (>24 hours) patients with illness onset from 25 May to 1 July 2009	Population controls recruited through random-digit-dial to homes proportionate to number of cases per age group and region	**Non-hospitalized:** 384**Hospitalized:**270	**Community controls:**603	OR	Age, comorbidity, sex, HCW status	Hospitalized compared to nonhospitalized cases; 2007–08 TIV receipt; TIV receipt prior 5 y; age (50 y; 60 y) and comorbidity stratified.
**Ontario test-negative case-control**	Specimens submitted to Ontario Provincial Laboratory or Mount Sinai Hospital/University Health Network (Toronto)	Household telephone survey of cases and controls conducted 1 August to 4 September 2009	Medically attended pH1N1 test-positive non-hospitalized and hospitalized patients with specimen submitted for influenza testing from 13 April to 20 July 2009	Medically attended pH1N1 test-negative non-hospitalized and hospitalized patients with specimen submitted for influenza testing from 13 April 1to 20 July 2009	**Non-hospitalized:**250**Hospitalized:**136	**Test-negative controls:**288	OR	Age, comorbidity, sex, HCW status, specimen collection date (before/after 11 June), prior physician visits in past 12 mo. Number of children in household also explored.	Hospitalized compared to nonhospitalized cases; 2007–08 TIV receipt; TIV receipt prior 5 y; age (50 y) and comorbidity stratified. Restriction based on test date within 4 d of symptom onset.
**Quebec household transmission study (prospective cohort)**	Quebec City, Quebec, Canada	Study conducted 27 May to 10 July 2009	Secondary attack rates for respiratory symptoms, influenza-like illness and laboratory-confirmed pH1N1 in household members of confirmed pH1N1 case compared for vaccinated and unvaccinated children (<18 y) and adults (≥18 y).		47 households participated with information on secondary attack rates collected from 120 household members: 32 received 2008–09 TIV and 88 did not receive 2008–09 TIV; 42 had confirmed pH1N1 and 78 tested negative.		RR	Stratified for children (<18 y) and adults (≥18 y).	Sex, comorbidity and severity indicators (median number of days of interference with daily activities or bedridden) and date to first specimen collection compared.

### Ethics Statement

The studies described were approved by the relevant institutional review boards of participating agencies/provinces as well as nationally for the sentinel study, or were conducted as legally mandated public health investigations for the Quebec and Ontario case-control studies. Patients provided written informed consent for the prospective cohort study and oral consent for the other three studies.

### National Sentinel Monitoring System

The four most populated provinces of Canada—BC, Alberta, Ontario, and Quebec—conduct annual (November through April) vaccine effectiveness (VE) monitoring through linked community-based sentinel physician surveillance networks. Study methods, implemented annually since 2004 and described in detail in prior publications, are approved each year by ethics boards provincially and nationally [10]–[12]. Designated sentinel sites in participating provinces are provided kits with which clinicians submit respiratory specimens (nasal or nasopharyngeal [NP]) for influenza testing along with epidemiologic information collected in a standardized way from eligible consenting patients presenting within 7 days of onset of influenza-like illness (ILI). Following the emergence of pH1N1 in mid-April, sentinel physicians in participating provinces were instructed to continue their routine VE monitoring activities, regardless of other testing guidelines to clinicians.

Seasonal influenza analysis spanned ILI onset between 1 November 2008 and 31 March 2009. pH1N1 analysis spanned ILI onset between 17 April and 22 July 2009. Testing for seasonal influenza at designated provincial laboratories in BC, Ontario, and Quebec consisted of real time reverse transcription PCR (RT-PCR) screening for influenza A and B, followed by real time RT-PCR subtyping (A/H1 or A/H3). In Alberta, all specimens were tested by Luminex RVP Assay, which detects seasonal influenza, pH1N1, and influenza B viruses and directly subtypes as A/H1 or A/H3. Testing was modified in all provinces after 17 April to detect pH1N1 using a conventional (end point) RT-PCR assay (Text S1, Appendix A1). Sequence analysis was performed on a subset of the PCR product in each province to validate specificity of pH1N1 detection.

We used the test-negative case-control design: cases presented with ILI and tested positive for seasonal or pH1N1 influenza, depending upon the analysis period; controls presented with ILI and tested negative for both seasonal and pH1N1 influenza. Medically attended, laboratory-confirmed influenza is uncommon, and thus case subjects are a small fraction of the total source population. In addition, control subjects are a proportion of the total population that reflects vaccine coverage and is anticipated to be stable over both the seasonal and the pH1N1 analysis periods. In that context, the odds ratio (OR) is appropriate to estimate the risk ratio (RR) [13]. Logistic regression was thus used to estimate ORs for a 2008–09 TIV effect on laboratory-confirmed influenza through sequential models with adjustment for age, chronic conditions, province, and timeliness of medical visit (sex was also explored but was not influential in final models). Per above, stratification at age 50 was decided *a priori* because of age-related differences generally observed in the epidemiology of pH1N1. VE is typically calculated as 1 – OR; here, we report ORs directly [14]. Additional methodological detail, including laboratory testing procedures, is provided in Text S1, Appendix A1.

### Quebec Population Case-Control Study

A legally mandated public health investigation was launched in Quebec on 15 July 2009. Eligible cases included community (medically attended, nonhospitalized) and hospitalized (≥24 hours) laboratory-confirmed detections of pH1N1 illness with onset from 25 May to 1 July among residents of one of four regions of Quebec together comprising 83% of community cases and 78% of hospitalized cases in Quebec during that period. All tests for pH1N1 were conducted at the provincial laboratory by specific RT-PCR assay. The study sampling plan included all hospitalized cases, approximately the same number of community cases and a number of controls similar to the sum of the hospitalized and community cases. The initial study plan was to use a test-negative case-control approach. Results from this test-negative approach was consistent with increased risk of pH1N1 illness among prior TIV recipients, but more in-depth examination of participant profiles showed test-negative specimens to be inadequate as controls for reasons outlined in Text S2, Appendix B1a–B1e, notably because of early restrictions placed on testing in Quebec. Quebec therefore moved to conventional sampling of population controls from the community. Population controls were selected by random-digit-dial to homes in numbers proportionate to the number of cases per age group and region. Trained interviewers conducted the survey from 17 July to 10 August 2009 using a standardized questionnaire. ORs were derived through sequential logistic regression for a 2008–09 TIV effect on pH1N1 cases compared to community controls with adjustment for relevant covariates (Tables 1 and 5).

### Ontario Test-Negative Case-Control Study

An urgent public health investigation was also conducted in Ontario based on test-negative case-control design. A sample size of 300 cases and 600 controls was estimated to be required *a priori* based on TIV prevalence in controls of 30%–35%, an alpha value of 0.05, and statistical power of 80% to detect an OR of at least 1.5 for increased risk of medically attended pH1N1 illness. Respiratory specimens (nasal, throat, or NP swabs) submitted for influenza testing between 13 April and 20 July 2009 at the provincial laboratory or the Mount Sinai Hospital/University Health Network were identified as representing a community (medically attended, non-hospitalized) or hospitalized case (pH1N1-specific RT-PCR positive) or a control (RT-PCR negative for all influenza). Hospitalized cases were identified through linkage to the public health surveillance database. Test-negative controls were medically attended community-based patients. A sample of cases and controls was randomly selected and frequency-matched by age group and week of specimen collection. A standardized telephone survey of consenting cases and controls was administered between 1 August and 4 September 2009 by trained interviewers from the Institute for Social Research (ISR), York University, Toronto. In the analysis, test date before versus after 11 June was incorporated as a binary variable into all models since Ontario clinicians (outside the sentinel system) were instructed after that date to submit specimens only from high-risk patients and/or patients with severe illness. ORs were derived through sequential logistic regression for a 2008–09 TIV effect on pH1N1 cases compared to test-negative community controls with adjustment for relevant covariates (Tables 1 and 5).

### Quebec Household Transmission Study

A prospective study to assess household pH1N1 transmission and secondary attack rates (SARs) was conducted in Quebec City, Canada from 27 May to 10 July 2009. The effect of TIV on pH1N1 risk was thus also explored in this cohort. The household primary case was the first laboratory-confirmed patient to present with symptoms of respiratory illness in the household. Primary cases were excluded as were symptomatic household members without laboratory-confirmation of pH1N1 whose symptom onset preceded that of the primary case. NP swabs were collected at home from all household members as soon as possible after identification of the primary case. Clinical information and self-reported 2008–09 TIV status were collected using a standardized questionnaire. NP swabs were tested by RT-PCR assay. SARs were compared across vaccination groups by Chi-square (or Fisher exact as appropriate) for children (<18 years) and adults (≥18 years), and RRs were computed.

## Results

### National Sentinel Monitoring System

Inclusion/exclusion criteria and final participation in the seasonal and pH1N1 sentinel study analysis periods are shown in Text S1, Appendix A2. Influenza subtype and pH1N1 detection by province are shown in Text S1, Appendix A3a/b, and the epidemic curve of influenza test results by subtype and week of ILI onset is shown in Text S1, Appendix A4. There were 1,529 specimens included in the overall seasonal analysis, of which 672 (44%) were influenza positive; by subtype these included 253 A/H3 (38%), 249 B (38%), and 160 A/H1 (24%). Ten were non-subtypeable. There were 711 specimens included in pH1N1 analyses, of which 175 (25%) were influenza positive, 144 (82%) for pH1N1 (20% pH1N1 positivity), 29 (17%) for seasonal influenza, and two of unknown subtype. Seasonal influenza viruses were detected in 28 sentinel specimens with ILI onset between 20 April and 10 May 2009, including 14 A/H3 (50%), 11 B (39%), and three A/H1 (11%); A/H1 was detected in one specimen thereafter.

The profile of participants is comparable between seasonal and pH1N1 analysis periods (Table 2; more detail in Text S1, Appendix A5a/b). Young adults aged 20–49 years contributed most to the sentinel study during both seasonal and pH1N1 analysis periods. In participating provinces, 9% of the general population 12–19 years, and 10% aged 20–49 years, are estimated from publicly available data to have vaccine-eligible chronic conditions [15]. These proportions are comparable to those found among seasonal and pH1N1 test-negative controls in the sentinel study. Compared to population estimates of ∼20%–25% for 50- to 64-year-olds and ∼35%–40% for those ≥65 years, the report of chronic conditions was substantially higher among older adult controls for both seasonal (64/172 [37%] and 57/85 [67%], respectively) and pH1N1 (34/109 [31%] and 27/48 [56%]) analysis periods (Text S1, AppendixA5a/A5b) [15]. The proportion immunized in 2008–09 among test-negative controls was comparable for seasonal and pH1N1 analysis periods both overall (287/857 [33%] and 166/536 [31%], respectively) and among controls ages 20–49 years (86/384 [22%] and 63/272 [23%], respectively). Overall, immunization coverage estimates among controls were similar to those derived from the 2007–08 Canadian Community Health Survey (CCHS), particularly among young adults (Text S3, Appendix C) [16].

**Table 2:** Sentinel test-negative case-control study: Participant profile by analysis period (seasonal influenza and pH1N1).

Participant Attribute Category	Subcategory	Seasonal Influenza Analysis Period (ILI Onset: 1 November 2008 to 31 March 2009)			pH1N1 Analysis Period (ILI Onset: 17 April 2009 to 22 July 2009)		
		Cases (N = 672), n/N (%)	Controls (N = 857), n/N (%)	ALL (N = 1,529), n/N(%)	Cases (N = 144), n/N (%)	Controls (N = 536), n/N (%)	ALL (N = 680), n/N(%)
**2008–09 TIV Receipt**		97 (14)	287 (33)	384 (25)	45 (31)	166 (31)	211 (31)
**Age (y)**	1–8	100 (15)	93 (11)	193 (13)	13 (9)	41 (8)	54 (8)
	9–19	176 (26)	123 (14)	299 (20)	59 (41)	66 (12)	125 (18)
	20–49	311 (46)	384 (45)	695 (45)	59 (41)	272 (51)	331 (49)
	50–64	69 (10)	172 (20)	241 (16)	10 (7)	109 (20)	119 (18)
	≥65	16 (2)	85 (10)	101 (7)	3 (2)	48 (9)	51 (8)
	Median (range), y	23 (1–85)	38 (1–92)	30 (1–92)	20 (2–68)	38 (1–92)	33 (1–92)
**2008–09 TIV immunized by age group**	1–8	14 (14)	20 (22)	34 (18)	5 (38)	4 (10)	9 (17)
	9–19	14 (8)	23 (19)	37 (12)	13 (22)	6 (9)	19 (15)
	20–49	39 (12)	86 (22)	125 (18)	22 (37)	63 (23)	85 (26)
	50–64	17 (25)	84 (49)	101 (42)	2 (20)	60 (55)	62 (52)
	≥65	13 (81)	74 (87)	87 (86)	3 (100)	33 (69)	36 (71)
**Sex**	Female	371 (55)	503 (59)	874 (57)	79 (55)	332 (62)	411 (60)
**Interval between ILI onset and specimen collection**	≤4 d	569 (85)	651 (76)	1220 (80)	128 (89)	418 (78)	546 (80)
	5–7 d	103 (15)	206 (24)	309 (20)	16 (11)	118 (22)	134 (20)
	Median (range)	3 (0–7)	3 (0–7)	3 (0–7)	2 (0–7)	3 (0–7)	3 (0–7)
**With chronic conditions**	N	89 (13)	199 (23)	288 (19)	23 (16)	102 (19)	125 (18)
	Vaccinated 2008–09	37 (42)	133 (67)	170 (59)	14 (61)	61 (60)	75 (60)
**Without chronic conditions**	N	583 (87)	658 (77)	1241 (81)	121 (84)	434 (81)	555 (82)
	Vaccinated 2008–09	60 (10)	154 (23)	214 (17)	31 (26)	105 (24)	136 (24)
**Province**	Alberta	146 (22)	375 (44)	521 (34)	27 (19)	167 (31)	194 (29)
	BC	191 (28)	189 (22)	380 (25)	19 (13)	166 (31)	185 (27)
	Ontario	126 (19)	166 (19)	292 (19)	34 (24)	102 (19)	136 (20)
	Quebec	209 (31)	127 (15)	336 (22)	64 (44)	101 (19)	165 (24)
**Chronic condition by province**	Alberta	19 (13)	99 (26)	118 (23)	6 (22)	34 (20)	39 (20)
	BC	27 (14)	40 (21)	67 (18)	6 (32)	39 (23)	45 (24)
	Ontario	20 (16)	33 (20)	53 (18)	4 (12)	10 (10)	14 (10)
	Quebec	23 (11)	27 (21)	50 (15)	7 (11)	19 (19)	26 (16)
**2008–09 TIV immunized by province**	Alberta	15 (10)	113 (30)	128 (25)	9 (33)	56 (34)	65 (34)
	BC	20 (10)	55 (29)	75 (20)	4 (21)	46 (28)	50 (27)
	Ontario	32 (25)	75 (45)	107 (37)	11 (32)	37 (36)	48 (35)
	Quebec	30 (14)	44 (35)	74 (22)	21 (33)	27 (27)	48 (29)
**2007–08 TIV immunized by province** a	Alberta	28 (20)	121 (33)	149 (29)	8 (30)	52 (33)	52 (28)
	Ontario	47 (39)	80 (51)	127 (46)	14 (48)	41 (41)	41 (32)
	Quebec	33 (16)	40 (33)	73 (22)	18 (31)	28 (29)	28 (18)

a BC did not collect information on 2007–08 vaccine status. 2007–08 percentages derived based on participants for whom vaccine status known. Denominators do not include those with missing information (never exceeding four missing counts for any age category).

**Figure 1:**
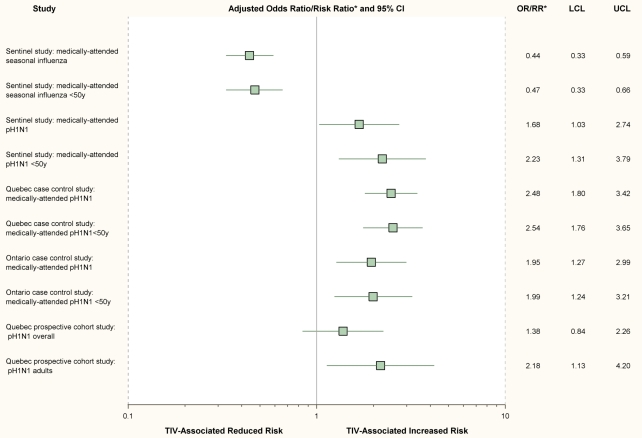
Summary of main findings. Fully adjusted effect measures and 95% confidence intervals from four epidemiologic studies in Canada to assess the association between 2008–09 trivalent inactivated influenza vaccine (TIV) and risk of community-based seasonal influenza and pH1N1 illness. *Note: Boxes show point estimates for the OR for each study/subgroup except the Quebec prospective cohort study for which the effect measure displayed is the RR. See Tables 1, 3, and 5 for covariates included in adjusted analyses. RR for the Quebec prospective cohort study was age-stratified but not further adjusted (see tables 1 and 6). LCL, lower confidence limit; UCL, upper confidence limit.

As in previous seasons, we found that prior vaccination with TIV significantly reduced the risk of medically attended seasonal influenza in 2008–09, with ORs consistently and significantly <1 (Figure 1, Table 3). Conversely, receipt of TIV was associated with significantly increased risk of medically attended pH1N1 illness with fully adjusted OR (95% CI) of 1.68 (1.03–2.74) overall and 2.23 (1.31–3.79) for participants <50 years (Figure 1, Table 3). Fully adjusted sensitivity analyses, further restricted to participants 20–49 years of age, to those with ILI onset after mid-May (the period of greatest indigenous pH1N1 circulation), or to the provinces of Quebec and Ontario (more intense pH1N1 activity) gave consistent results (Table 3). ORs were highest for a vaccine effect on pH1N1 risk in Quebec. Caution is required in the interpretation of a TIV effect for the age stratum >50 years owing to small sample size, wide confidence intervals spanning 1, and further anticipated variation of effect with advancing age and immune status among older individuals [17],[18]. Estimates for crude and fully adjusted ORs for 2008–09 TIV effect on seasonal influenza for the period 17 April to 30 May 2009 remained <1, although sample size was again small and statistical significance was not achieved. In overall analyses, the crude and fully adjusted ORs for 2008–09 TIV in preventing seasonal influenza during the pH1N1 analysis period 17 April to 30 May were 0.48 (0.18–1.30) and 0.73 (0.25–2.16), respectively, and for the age stratum <50 years were 0.55 (0.16–1.93) and 0.87 (0.23–3.32), respectively. Crude and adjusted ORs for TIV effect on seasonal influenza were also <1 for the full pH1N1 analysis period spanning 17 April to 22 July.

**Table 3:** Sentinel test-negative case-control study: ORs (95% CIs) for 2008–09 TIV effect on seasonal and pH1N1 illness overall and stratified by age, with adjustment for relevant covariates, and with additional restrictions as specified.

Covariates	Overall		Age <50 Years		Age ≥50 Years	
	Seasonal Influenza, N = 1,529	pH1N1^a^, N = 680	Seasonal Influenza, N = 1,187	pH1N1^a^, N = 510	Seasonal Influenza, N = 342	pH1N1^a^, N = 170
Unadjusted	0.33 (0.26–0.43)	1.01 (0.68–1.51)	0.47 (0.34–0.65)	1.84 (1.17–2.89)	0.34 (0.20–0.57)	0.43 (0.13–1.37)
Chronic conditions (yes/no)	0.37 (0.28–0.48)	1.07 (0.71–1.62)	0.47 (0.34–0.66)	1.85 (1.16–2.96)	0.37 (0.22–0.64)	0.40 (0.12–1.31)
Age^b^	0.46 (0.35–0.60)	1.87 (1.19–2.94)	0.48 (0.35–0.67)	2.39 (1.47–3.89)	0.38 (0.22–0.67)	0.44 (0.14–1.45)
Province (BC, AB, ON, QC)	0.32 (0.24–0.42)	1.01 (0.67–1.53)	0.47 (0.34–0.66)	1.85 (1.15–2.97)	0.30 (0.17–0.52)	0.43 (0.13–1.42)
Interval since ILI onset (≤4 d/>4 d)	0.34 (0.26–0.44)	1.07 (0.72–1.60)	0.47 (0.34–0.65)	1.91 (1.21–3.00)	0.34 (0.20–0.57)	0.42 (0.13–1.37)
Age + chronic conditions	0.46 (0.35–0.62)	1.75 (1.10–2.79)	0.48 (0.34–0.67)	2.27 (1.37–3.76)	0.40 (0.23–0.71)	0.42 (0.13–1.39)
Age+chronic conditions+province	0.44 (0.33–0.60)	1.62 (1.00–2.63)	0.47 (0.33–0.66)	2.16 (1.28–3.65)	0.34 (0.19–0.62)	0.41 (0.12–1.41)
Age+chronic conditions+province+ interval	0.44 (0.33–0.59)	1.68 (1.03–2.74)	0.47 (0.33–0.66)	2.23 (1.31–3.79)	0.33 (0.18–0.61)	0.42 (0.12–1.46)
**Adjusted estimates by specification**						
Restricted to period 17 May to 22 Julyb,c	NA	1.69 (0.94–3.02)	NA	2.45 (1.28–4.71)	NA	NSS
Restricted to Quebec and Ontario onlyb,c	0.48 (0.32–0.72)	2.15 (1.14–4.04)	0.49 (0.30–0.80)	3.03 (1.54–5.97)	NSS	NSS
Restricted to Quebec onlyb,c	0.49 (0.27–0.90)	2.66 (1.15–6.18)	0.61 (0.28–1.32)	4.50 (1.74–11.69)	NSS	NSS
Restricted to Ontario onlyb,c	0.48 (0.27–0.83)	1.67 (0.65–4.31)^†^	0.47 (0.24–0.90)	2.04 (0.73–5.70)	NSS	NSS
Restricted to adults 20–49 y onlyb,c	NA	NA	0.43 (0.28–0.68)	2.20 (1.16–4.18)	NA	NA
Restricted only to those with no chronic conditionsb,c	0.43 (0.30–0.61)	1.48 (0.87–2.50)	0.42 (0.29–0.61)	2.39 (1.34–4.29)	NSS	NSS
**Vaccine status definition**						
Immunized in 2007–08b,c,d (±2008–09)	0.72 (0.52–0.99)	1.58 (0.93–2.71)	0.79 (0.54–1.14)	1.89 (1.06–3.38)	NSS	NSS
Immunized in 2007–08 but not 2008–09b,c,d	1.00 (0.63–1.60)	1.48 (0.63–3.44)	1.00 (0.60–1.66)	1.33 (0.54–3.23)	NSS	NSS
Immunized in 2008–09 but not 2007–08b,c,d	0.23 (0.10–0.52)	1.91 (0.72–5.05)	0.17 (0.06–0.47)	2.06 (0.70–6.02)	NSS	NSS
Immunized in 2007–08 and 2008–09 b,c,d	0.47 (0.32–0.69)	1.65 (0.92–2.95)	0.54 (0.34–0.86)	2.18 (1.15–4.14)	NSS	NSS

a Specimens positive for seasonal influenza (29) or of unknown subtype (2) were excluded as controls from pH1N1 analysis period.

b Adjusted for age as 1–8, 9–19, 20–49, 50–64, and ≥65 y, where possible, and 1–8, 9–49, ≥50 y where zero cells preclude adjustment with finer age categories (indicated by †); not further age adjusted for 20–49 years. Referent age category was the last age group included in each analysis, e.g., for overall analyses it was the category ≥65 y, and for the analysis restricted to those <50 y, it was the category 20–49 y.

c Fully adjusted includes chronic conditions (yes/no), age, province (as appropriate), and interval since ILI onset (≤4 d/>4 d);

d Includes data only from Alberta, Ontario and Quebec; BC did not collect information on 2007–08 immunization status. Children <3 y excluded from these analyses.

NA, not applicable; NSS, insufficient sample size precluding reliable estimation.

### Quebec Population Case-Control Study

Among community members who answered the telephone survey as potential controls, the participation rate before applying exclusions was 62% (630/1,014) for the Quebec case-control study (details provided in Text S4, Appendix D1). After exclusion criteria were applied, 384 community cases, 270 hospitalized cases, and 603 community controls were available for the Quebec case-control analysis (Text S4, Appendix D1). Participant profiles are shown in Table 4 and in more detail in Text S4, Appendix D2. In community controls aged 12–19, 20–34, 35–44, and 45–64 years, TIV coverage for the 2008–09 season were 6% (8/125), 16% (16/101), 18% (13/71), and 24% (35/144), respectively, similar to estimates from the CCHS for Quebec as measured for the 2007–08 season (Text S3, Appendix C) [16]. The proportion of controls with underlying medical conditions eligible for TIV was also comparable to population estimates by age (Text S4, Appendix D2) [15].

**Table 4:** Quebec and Ontario pH1N1 case-control studies: Participant profiles.

Participant Attribute	Subcategory	QUEBEC STUDY			ONTARIO STUDY		
		Community Cases, n (%)	Hospitalized Cases, n (%)	Community Controls, n (%)	Community Cases, n (%)	Hospitalized Cases, n (%)	pH1N1-negative controls, n (%)
**N**		384	270	603	250	136	295
**Age**	6–59 mo	12 (3)	35 (13)	40 (7)	17 (7)	20 (15)	27 (9)
	5–19 y	158 (41)	73 (27)	201 (33)	134 (54)	57 (42)	72 (24)
	20–34 y	89 (23)	40 (15)	101 (17)	30 (12)	26 (19)	58 (20)
	35–49 y	62 (16)	50 (18)	114 (19)	36 (14)	17 (13)	67 (23)
	50–59 y	44 (11)	37 (14)	88 (15)	24 (10)	8 (6)	41 (14)
	≥60 y	19 (5)	35 (13)	59 (10)	9 (4)	8 (6)	30 (10)
	Median (range)	23 (1–84) y	28 (1–95) y	29 (1–96) y	15 (1–77) y	16 y (8 mo to 85 y)	32 (1–85) y
**Symptoms**	Fever+cough	270 (70)	218 (59)	18 (3)	167 (67)	97 (71)	109 (41)
	ILI	261 (68)	159 (59)	15 (2)	166 (66)	93 (68)	107 (40)
**Female**		217 (56)	144 (53)	353 (59)	155 (62)	94 (69)	188 (65)
**Comorbidity** a		96 (25)	164 (61)	95 (16)	35 (14)	51 (38)	51 (18)
**Smoker** b ^,^ c		43 (11)	47 (17)	85 (14)	21 (18)	20 (30)	61 (21)
**HCW** d		68 (18)	8 (3)	37 (6)	17 (7)	8 (6)	23 (8)
**Median physician visits prior year**		N/A	N/A	N/A	3	4	3
**Median number of children in household**		N/A	N/A	N/A	1	1	0
**TIV status**	2008–09	145 (38)	119 (44)	119 (20)	109 (44)	46 (34)	105 (36)
	2007–08^c^	151 (40)	120 (47)	132 (22)	124 (50)	64 (47)	119 (41)
	2007–08^c^ only	29 (8)	19 (7)	34 (6)	28 (11)	22 (16)	35 (19)
	2008–09 only	23 (6)	18 (7)	20 (3)	13 (5)	4 (3)	19 (11)
	2007 and 2008^c^	122 (32)	99 (39)	98 (16)	96 (38)	42 (31)	84 (29)
	5 in 5 y^c^	80 (22)	74 (32)	71 (13)	69 (30)	34 (30)	60 (20)
	≥1 in 5 y	198 (52)	154 (57)	182 (30)	164 (66)	88 (65)	171 (58)
	None in 5 y	182 (47)	94 (35)	421 (70)	76 (33)	35 (30)	124 (42)
	1–3 in 5 y^c^	88 (24)	38 (17)	75 (14)	68 (29)	34 (30)	93 (32)
	4–5 in 5 y^c^	101 (27)	83 (36)	85 (15)	89 (38)	46 (40)	78 (26)
	No comorbidity (N)	288	106	508	207	83	231
	TIV 2008–09	99 (34)	27 (25)	83 (16)	90 (43)	23 (28)	75 (32)
	With comorbidity (N)	96	164	95	35	51	51
	TIV 2008–09	46 (48)	92 (56)	36 (38)	16 (46)	23 (45)	24 (47)

NOTE: Where percentages do not sum 100%, this is due to missing data. ILI is defined as fever+cough+one or more of: sore throat, myalgia, arthralgia, headache, or prostration.

a Comorbidity or chronic condition is defined as a vaccine-eligible underlying condition.

b Smoker is defined as any current smoking.

c For these variables, denominators are slightly different because of missing or not applicable values.

d HCW is defined as any person working in the health care system with or without direct contact with patients.

N/A, not available.

The crude OR for a 2008–09 TIV effect on pH1N1 community cases was significant compared to controls (2.47 [1.85–3.30]) (Table 5). Crude OR was also significant for hospitalized (3.20 [2.34–4.38]) cases (Table 5). After adjustment for age, chronic conditions, sex, and HCW status, the OR for community cases was 2.48 (1.80–3.42) and for hospitalized cases was 2.16 (1.47–3.17) (Figure 1, Table 5). TIV receipt did not appear to increase the risk when comparing hospitalized to community cases (adjusted OR of 0.97 [0.66–1.42]).

**Table 5:** Quebec and Ontario case-control studies: ORs (95% CI) for TIV effect on pH1N1.

OR for Previous 2008–09 TIV	QUEBEC STUDY			ONTARIO STUDY		
	Community Cases Versus Community Controls, N = 384/603	Hospitalized Cases Versus Community Controls, N = 270/603	Hospitalized Cases Versus Community Cases, N = 270/384	Community Cases Versus Test-Negative Community Controls, N = 250/288	Hospitalized Cases Versus Test-Negative Community Controls, N = 136/288	Hospitalized Cases Versus Community Cases, N = 136/250
**Crude**	2.47 (1.85–3.30)	3.20 (2.34–4.38)	1.30 (0.94–1.78)	1.56 (1.07–2.27)	1.03 (0.58–1.82)	0.67 (0.42–1.06)
**Adjusted for:**						
Age (ref: 20–34 y)	3.05 (2.24–4.14)	3.22 (2.31–4.50)	1.06 (0.76–1.50)	1.93 (1.28–2.90)	1.37 (0.74–2.54)	0.68 (0.42–1.09)
Comorbidity (chronic condition)	2.32 (1.73–3.10)	1.90 (1.33–2.72)	0.94 (0.66–1.33)	1.68 (1.14–2.46)	0.94 (0.52–1.71)	0.62 (0.38–1.00)
Sex (ref: male)	2.51 (1.88–3.35)	3.31 (2.41–4.54)	1.32 (0.96–1.82)	1.55 (1.07–2.26)	1.03 (0.58–1.82)	0.67 (0.42–1.05)
HCW	2.16 (1.61–2.91)	3.31 (2.41–4.54)	1.60 (1.14–2.22)	1.62 (1.10–2.37)	1.09 (0.61–1.95)	0.67 (0.43–1.07)
Physician visits^a^				1.60 (1.08–2.37)	1.03 (0.56–1.88)	0.65 (0.41–1.06)
Age+comorbidity	2.86 (2.10–3.90)	2.07 (1.42–3.03)	0.80 (0.55–1.16)	2.06 (1.35–3.13)	1.21 (0.63–2.30)	0.67 (0.41–1.11)
Age+comorbidity+sex	2.90 (2.12–3.96)	2.12 (1.45–3.10)	0.82 (0.56–1.18)	2.04 (1.34–3.12)	1.23 (0.64–2.36)	0.67 (0.40–1.10)
Age+comorbidity+sex+HCW	2.48 (1.80–3.42)	2.16 (1.47–3.17)	0.97 (0.66–1.42)	1.95 (1.27–2.99)	1.19 (0.61–2.32)	0.68 (0.41–1.13)
Age+comorbidity+sex+physician visits	N/A	N/A	N/A	2.08 (1.33–3.26)	1.22 (0.60–2.47)	0.67 (0.39–1.13)
**Adjusted** b **OR by category of seasonal influenza immunization in persons aged ≥5 y:**						
Immunized in 2007–2008	2.55 (1.84–3.54)	2.19 (1.47–3.26)	1.01 (0.68–1.53)	1.98 (1.28–3.06)	3.17 (1.56–6.46)	1.20 (0.71–2.05)
Immunized in 2007–08 and 2008–09	2.55 (1.79–3.63)	2.26 (1.47–3.47)	1.01 (0.66–1.54)	2.32 (1.43–3.75)	2.09 (0.99–4.40)	0.92 (0.53–1.59)
≥1 in last 5 y versus never	2.84 (2.08–3.87)	2.52 (1.70–3.73)	1.09 (0.73–1.64)	1.95 (1.26–3.03)	2.80 (1.34–5.82)	1.33 (0.76–2.33)
5 in last 5 y versus never	2.77 (1.80–4.28)	3.24 (1.97–5.34)	1.42 (0.86–2.37)	1.81 (1.09–3.02)	2.03 (0.93–4.43)	0.92 (0.52–1.65)
**Adjusted** b **OR by number of vaccinations in prior 5 y in persons aged ≥5 y**						
0	Reference	Reference	Reference	Reference	Reference	Reference
1, 2, 3	2.56 (1.75–3.74)	1.21 (0.72–2.01)	0.68 (0.41–1.13)	1.56 (0.94–2.57)	2.22 (0.98–5.02)	1.35 (0.70–2.61)
4, 5	2.92 (1.98–4.30)	2.17 (1.38–3.41)	0.90 (0.57–1.43)	2.57 (1.51–4.38)	3.80 (1.60–8.99)	1.32 (0.70–2.49)
**Adjusted** c **OR by strata**						
No comorbidity	2.71 (1.87–3.93)	1.77 (1.04–2.99)	0.68 (0.38–1.19)	2.46 (1.52–3.99)	1.77 (0.78–4.07)	0.53 (0.29–0.98)
With comorbidity^d^	1.72 (0.87–3.38)	2.93 (1.61–5.36)	1.47 (0.84–2.58)	0.80 (0.28–2.26)	0.64 (0.20–2.08)	1.53 (0.53–4.44)
**Age <50 y** e	2.54 (1.76–3.65)	2.27 (1.44–3.58)	1.00 (0.64–1.51)	1.99 (1.24–3.21)	1.23 (0.59–2.57)	0.63 (0.37–1.08)
**Age ≥50 y** d ^,^ f	2.03 (1.04–3.96)	2.06 (1.03–4.13)	1.25 (0.55–2.84)	1.89 (0.73–4.91)	1.21 (0.24–6.18)	1.29 (0.26–6.50)
**Age 50–59 y** g	4.23 (1.81–9.92)	1.48 (0.58–3.76)	0.45 (0.14–1.38)	N/A	N/A	N/A
**Age 60 y+** h	0.66 (0.22–2.01)	3.32 (1.06–9.74)	5.36 (1.44–19.95)	N/A	N/A	N/A

Ontario Study: Test date was included as a binary variable (before/after June 11, 2009) in all Ontario models (see text). ^a^Physician visits = number of visits to family doctor/primary care physician in the 12 mo prior.

b Adjusted for age (5–19, 20–34 [as reference], 35–49, 50–59, and 60+ y)+comorbidity+sex+HCW.

c Adjusted for age+sex+HCW.

d In Ontario adjustment for HCW not performed if not represented in all cells.

e Adjusted for age (<10, 10–19 y, and 20–49 [as reference])+comorbidity+sex+HCW.

f Adjusted for age (50–59 [as reference] and 60+ y)+comorbidity+sex+HCW.

g Adjusted for chronic condition+sex+HCW.

h Adjusted for comorbidity+sex.

N/A, not available.

### Ontario Test-Negative Case-Control Study

Text S5, Appendix E1 outlines inclusion and exclusion criteria resulting in 250 community cases, 136 hospitalized cases, and 288 test-negative community controls available for analysis in the Ontario test-negative case-control study. Baseline characteristics of community or hospitalized cases and controls are shown in Table 4 and in more detail in Text S5, Appendix E2. Community and hospitalized cases were younger than controls but reported similar health care–seeking behavior as measured by the median number of physician visits in the prior 12 months (Table 4). Among participants reporting any “flu-like” symptoms at the time of influenza testing, 72% (166/230) of community and 73% (93/130) of hospitalized cases reported symptoms consistent with ILI, compared to 41% (106/256) of controls (59 participants were unable to recall specific symptoms). Among controls 20–49 years of age, 31% (38/123) had received TIV, slightly higher (by ∼5%-10%) compared to people 20–44 years from the 2007–08 CCHS for Ontario (Text S3, Appendix C) [16]. The proportion of controls aged 12–49 years with a chronic condition (18/143; 11%) was within the expected range based on available population estimates [15].

After adjustment for age, chronic conditions, sex, and HCW status, the OR for TIV effect on pH1N1 was 1.95 [1.27–2.99] for community cases compared to controls (Figure 1, Table 5). For hospitalized cases, the OR was 1.19 [0.61–2.32] (Table 5). Adjustment for neither the number of primary care visits in the previous 12 months (marker for health care–seeking behavior) nor number of children in the household (marker for exposure risk) had influence except to slightly increase ORs. Interval between symptom onset and specimen collection was available for 141/250 (56%) of the community cases and 171/229 (60%) of the test-negative controls. With restriction to only community cases and controls known to be tested within 4 days of symptom onset, the fully adjusted OR for TIV effect on pH1N1 increased to 2.37 (1.22–4.60). Restriction to only participants known to have had ILI gave consistent results, but with reduced power owing to small sample size. TIV receipt did not appear to increase the individual risk of hospitalization when hospitalized and community cases were compared (adjusted OR of 0.68 [0.41–1.13]).

### Household Transmission Study

Forty-seven households participated in the Quebec household transmission study. This included 47 primary cases, six members who were symptomatic before the primary case, and 120 other members. Among these 120 household members (median age 29 years, range 0–61 years), 32/120 (27%) had received the 2008–09 TIV, 8/48 (17%) of children and 24/72 (33%) of adults. Timeline to the first specimen collection after identification of the primary case was similar between vaccinated and unvaccinated participants at a median of 3 (range 1–14) versus 3 (range 1–16) days. The proportion of children with chronic conditions was 0% (0/8) for the vaccinated and 15% (6/40) for the unvaccinated, whereas for adults it was 21% (5/24) and 23% (11/48), respectively.

SARs by outcome category for vaccinated compared to unvaccinated children and adults are presented in Table 6. TIV was not associated with increased risk of nonspecific respiratory symptoms (*p* = 0.40) for any age category. No increased risk was found in children for any outcome category, but with only eight vaccinated pediatric participants, no definitive conclusions should be drawn. Among the larger sample of adults, TIV was associated with significantly increased risk for more specific outcomes of ILI syndrome (RR 3.20 [95% CI 1.17–8.74]) and laboratory-confirmed pH1N1 regardless of symptoms (RR 2.18 [1.13–4.20]). Results were essentially unchanged and remained significant with adjustment for sex. Comparison of syndrome severity among vaccinated versus unvaccinated laboratory-confirmed cases overall showed no significant differences, with similar mean number of days during which the patient was unable to carry on daily activities (2.7 versus 2.4) and during which the patient was bedridden (1.4 versus 1.5).

**Table 6:** Quebec household transmission study: Secondary pH1N1 attack rates among household members by 2008–09 TIV status.

Outcome Category and 2008–09 TIV Status	Children <18 y, N = 48	RR (95% CI) Vaccinated Versus Unvaccinated Children	Adults ≥18 y, N = 72	RR (95% CI) Vaccinated Versus Unvaccinated Adults	Total, N = 120	RR (95% CI) Vaccinated Versus Unvaccinated Overall
**Respiratory symptoms**						
Received TIV	4/8 (50%)	0.71 (0.35–1.47)	13/24 (54%)	0.96 (0.62–1.50)	17/32 (53%)	0.85 (0.59–1.22)
Did not receive TIV	28/40 (70%)	1	27/48 (56%)	1	55/88 (63%)	1
**Influenza-like illness (ILI) syndrome**						
Received TIV	3/8 (38%)	1.00 (0.38–2.66)	8/24 (33%)	3.20 (1.17–8.74)	11/32 (34%)	1.51 (0.82–2.80)
Did not receive TIV	15/40 (38%)	1	5/48 (10%)	1	20/88 (23%)	1
**Any PCR-confirmed pH1N1**						
Received TIV	2/8 (25%)	0.59 (0.17–2.06)	12/24 (50%)	2.18 (1.13–4.20)	14/32 (44%)	1.38 (0.84–2.26)
Did not receive TIV	17/40 (43%)	1	11/48 (23%)	1	28/88 (32%)	1

ILI is defined as fever + cough + one or more of sore throat, myalgia, arthralgia, headache, or prostration.

## Discussion

To assess the association between TIV and pH1N1 risk that was first identified in Canada during the late spring of 2009, several observational designs were pursued through the summer, a period when pH1N1 continued to circulate in Canada. As reported here, this included incident (sentinel network) and retrospective case-control methods with both test-negative and conventional community controls as well as a prospective cohort study. In this paper we report the expected finding that 2008–09 TIV was associated with a significant (56%) reduction in the risk of medically attended illness due to seasonal influenza. However, we also report the unexpected finding that TIV receipt was subsequently associated with a statistically significant (1.4- to 2.5-fold) increased risk of medically attended illness due to the novel pH1N1 virus (Figure 1). Because the latter result is contrary to established knowledge, greater scrutiny is required to determine whether these associations are more likely on balance to be real (causal) or due to a methodological flaw (bias). Reliance on observational methods means that even careful study design, implementation, and analysis cannot rule out the possibility of random variation (chance) or bias (selection, information) or confounding in explaining results. Consistency of results across different designs in various populations and with substantial sample sizes provides some reassurance against random variation, but the possible contributions of bias or confounding still warrant careful consideration.

Several methodological issues have thus been considered in assessing these findings. The sentinel test-negative approach provides estimates of TIV association with both seasonal and pH1N1 risk and offers several methodological advantages. The well-established and rehearsed sentinel system in Canada has consistently shown TIV protection against seasonal strains over the several years of its application, higher with better vaccine matches and lower with relative antigenic mismatches [10]–[12]. Canada's sentinel system has not previously found an increased risk of illness associated with TIV, even at the component-specific level in the context of substantial vaccine mismatch to circulating seasonal strains. With the emergence of the novel pH1N1 virus in mid-April we were able to extend routine 2008–09 sentinel monitoring activities into the spring and summer without changes in protocol. Canada has a publicly funded health care system that is free to patients at use, thus removing restrictions on access that may apply elsewhere. Sentinel physicians were specifically notified that they were exempt from limitations on respiratory specimen testing applied to other ambulatory care clinicians. Although patients may have themselves altered their usual patterns of physician visits, in accordance with protocol only submissions meeting the ILI definition were included in sentinel analyses, thereby standardizing in part, although not fully, for health care–seeking or illness severity. Influenza immunization registries do not exist in participating provinces, so all vaccine uptake estimates rely upon patient self-reports and cannot be further confirmed. Although imperfect recall may have introduced misclassification of vaccine status, immunization history was recorded by the clinician at the time of test submission, before the test result was known, thus helping to attenuate possible differential in recall by outcome. We also excluded submissions with unknown vaccine status. Unlike the Ontario and Quebec case-control studies, the sentinel approach did not collect information on occupation. Adjustment for HCW status, however, did not meaningfully influence ORs in the Ontario test-negative or Quebec population case-control studies, and when they were excluded in the Quebec test-negative approach, ORs were unchanged or slightly increased.

To further address the possible influence of health care–seeking behavior, we adjusted for the timeliness of medical visits in the sentinel and Ontario case-control studies and for chronic conditions in all studies. Comorbidity was analyzed as a dichotomous variable. Although all chronic conditions may not exert the same influence, further stratification on select categories of chronic conditions did not influence ORs in subanalyses (not shown; available upon request). It is possible that misclassification of comorbidity may have biased our results. If this effect exists in the sentinel study, however, it should be minor; underlying conditions were also recorded before test results were known, and our findings mainly reflect the experience of healthy young adults and school-aged children. It is important to note that subanalyses within the stratum of participants without any chronic conditions showed a consistent vaccine effect. The lower ORs and apparent absence of TIV effect on pH1N1 risk in the older stratum of sentinel study participants are noteworthy. During both analysis periods, the proportion of controls ≥50 years of age, and especially ≥65 years of age, who reported chronic conditions and who received TIV were higher than expected based on community surveys, flagging a possible concern with the controls in that stratum. Given the small sample size and the expected variation in important characteristics such as pre-existing pH1N1 immunity across advancing years of life, no specific conclusions can be drawn for older individuals based on these data [17],[18].

A strength of the sentinel study is that it enabled a direct comparison of participant profiles and vaccine effect using the same methods for seasonal and pH1N1 analysis periods. With similar methods and participant profiles for the two analysis periods, we found very different TIV associations with influenza risk, notably among younger persons. We found significant TIV protection against seasonal influenza and a protective trend against seasonal strains that persisted during the pH1N1 analysis period. ORs<1 for seasonal influenza that persisted after April 17 make it more difficult to attribute the ORs>1 for pH1N1 using the same methods during the same period to newly introduced bias.

With its randomly selected community controls, the Quebec population case-control study likely provides the upper bound of a risk estimate for pH1N1 associated with TIV, since standardization of health care–seeking behavior between cases and controls was not afforded. Results from this design may be affected by differential characteristics among those who sought care or testing compared to community controls. The initial Quebec experience with the test-negative case-control design also provides an important caution regarding the potential for selection bias with this approach. This caution applies especially to test-negative designs when specimens are drawn from general laboratory submissions and/or when limitations are placed on testing, such as to individuals with chronic conditions or severe illness (see Text S2, Appendix B1a). Careful assessment of participant profiles showed that the test-negative controls in the initial Quebec study were not adequately representative of the source population from which the cases arose, although with appropriate adjustment for relevant covariates and restriction to patients with similar clinical presentation, the same increased risk of pH1N1 illness in association with 2008–09 TIV was found (Text S2, Appendix B1a–B1e).

Ontario applied a later restriction on laboratory testing compared to Quebec (11 June versus 15 May), and the Ontario dataset showed test-negative controls to be representative of the case source population. Both cases and controls sought care and had similar health care–seeking behavior, as evidenced by the rate of physician visits in the previous 12 months and by the proportion with chronic conditions. Immunization rates among test-negative controls may have slightly exceeded population estimates for Ontario in young adults (tending to decrease the OR) but a statistically significant effect was still observed. Adjustment for other relevant covariates generally increased ORs among vaccinated participants. Sample sizes in Ontario were smaller than planned, especially for hospitalized cases, and confidence intervals surrounding ORs were wide, particularly among stratified analyses. Point estimates, however, remained consistent with other studies. A comparison between hospitalized and community cases in both the Quebec and the Ontario designs suggests that once pH1N1 illness was acquired, the risk of pH1N1 hospitalization was not further increased among TIV recipients. An increase in the absolute number of hospitalizations, however, would nevertheless still occur with this association, since that number is determined by the risk of becoming ill (increased) multiplied by the risk of hospitalization once ill (unchanged). Finally, with its active and prospective follow-up of all household members, the Quebec household transmission study provided corroborating evidence that was able to avert the potential for health care–seeking and other selection biases known to affect case-control studies. The results were consistent with other findings, and statistical significance was observed. A limitation of that study, however, is the small sample size that precluded further stratification.

Confounding by indication may spuriously lower VE estimates in observational studies because influenza vaccine is administered to people at greater risk of clinical illness and more likely to seek care (such as those with chronic conditions) [19]. More recent publications have also reported overestimation of influenza vaccine protection against serious outcomes among elderly persons as a result of better general health status among vaccinated compared to unvaccinated people (healthy user bias) [20],[21]. Influences on immunization status can thus skew estimates in either direction. It may be that in young people chronic conditions exert a greater influence in decreasing VE estimates (increasing ORs) than the counterforce of healthy user bias. Our national sentinel study population, however, included few participants with chronic conditions, and to further address this possible influence we applied recognized analysis techniques of restriction, stratification, and adjustment to all studies. We cannot rule out that these efforts were incomplete and that an unmeasured bias or residual confounding remains.

Several studies from Australia [22], Mexico [23],[24], and the US [25],[26] have instead reported null or protective effects of 2008–09 TIV against pH1N1 illness based on test-negative case-control [22]–[25], case-cohort [25], or ILI outbreak investigations [26]. One other test-negative case-control analysis from a US outbreak found a statistically significant increased risk, with an unadjusted OR of 2.9 (95% CI 1.8–4.69) for pH1N1 illness among military beneficiaries who received influenza vaccine within the previous 12 months [27]. Unlike in Canada, however, this association was driven primarily by receipt of live attenuated vaccine. Discrepant results across studies may reflect either differences in methods or real variation in the effect of specific vaccines, immunization programs, or population immunity. Most of the studies published to date, however, have not presented sufficient participant characteristics to properly assess methodological issues, sources of bias or confounding, or the validity of results. As previously highlighted, the importance of the public health and scientific implications requires that analyses of TIV effect on pH1N1 risk be more rigorous [28],[29]. At a minimum, a detailed profile of cases and controls specifically included in vaccine effectiveness analyses should be displayed by vaccine status, age, and chronic conditions, as well as timeliness of specimen collection and other recognized influences. Where participant characteristics have been presented by investigators, conspicuous evidence of selection bias can be seen to explain opposite findings of TIV protection against pH1N1 [23],[28]. In the detailed participant profiles we display for each of the four studies we report, evidence for this type of bias is not obvious, but that does not rule it out. We also cannot rule out the possibility that the increased risk of pH1N1 found in Canada was an effect specific to the Canadian vaccine: it is noteworthy that ORs were highest in Quebec, where a greater proportion of domestically produced vaccine is distributed than in the rest of Canada. However, even if our findings are considered a “Canadian problem,” if causal in nature they would still have wider implications for our understanding of influenza immunopathogenesis.

In the event that our findings are valid and reflect a causal association by which prior receipt of TIV increased the risk of PCR-positive pH1N1 illness, several biological mechanisms have been considered to explain them. These proposed mechanisms must each be viewed as speculative since our epidemiologic studies were not designed to assess them. One hypothesis is that repeat immunization effectively blocks the more robust, complex, and cross-protective immunity afforded by prior infection. This mechanism was suggested by Hoskins et al. in the mid-1970s in evaluating the risk of A/H3N2 drift variants among previously infected versus immunized boys during successive boarding school outbreaks, with the consideration of other factors (such as viral neuraminidase [NA]) in addition to HA antibody [30]. Bodewes et al. have more recently shown in mice that effective vaccination against human influenza A/H3N2 virus prevents virus-induced (cell-mediated) heterosubtypic immunity against severe (lethal) infection with avian influenza of a different subtype (A/H5N1) [31],[32]. That one influenza subtype may influence the risk of infection by another is also suggested by the subtype replacement that followed the pandemics of 1918, 1957, and 1968, although the mechanism for that is unknown [33]. A BLAST sequence analysis demonstrates that pH1N1 and human influenza strains (A/H1N1 and A/H3N2, recent [since 2000] and historical [since 1974]) are nearly identical (95%–98%) with respect to their polymerase proteins, at both the overall and the antigenic levels, and other internal components, including M1 (94%–97%) and NP (85%–92%) are also well-conserved (Text S6, Appendix F). Conversely, both the HA and the NA surface proteins of recently circulating seasonal H1N1 influenza viruses and pH1N1 are more divergent, particularly with respect to their antigenic regions (Text S6, Appendix F). With a greater likelihood of seasonal influenza infection, unvaccinated people may have had a greater opportunity to develop cross-protective cell-mediated immunity to the more conserved internal viral components of pH1N1. Since those immunized in a given season may have been repeatedly immunized over several seasons, they may have lost multiple opportunities for infection-induced cross-immunity. TIV recipients may have boosted antibody to HA/NA (the only TIV components), effectively protecting against seasonal influenza, but without a cross-protective effect against the markedly different surface antigens of pH1N1.

If this hypothesis explains our results, it also reassuringly implies that the TIV effect we have observed on pH1N1 risk would be induced again only if seasonal influenza circulates before pH1N1 and TIV blocks the potential cross-protection of that heterologous infection. There are, however, at least two considerations that oppose this hypothesis. First, the risk of laboratory-confirmed influenza illness per se—the outcome reported by our studies—is believed to be determined primarily by protective antibodies to viral surface proteins. As indicated above, the antigenic distance between the HA and NA surface proteins of pH1N1 and recently circulating human H1N1 strains is large, making cross-protection on that basis less likely. Differences in severe outcomes may be explained by cross-protective cell-mediated immunity, as shown by Bodewes et al. to be induced by prior infection with heterologous virus, but we did not detect differences in severe outcomes by vaccine status and that is not what we are trying to explain. The role of cell-mediated or other immune correlates of cross-protection against influenza illness per se likely warrants further study. Second, in order to show a 2-fold increase in pH1N1 illness, this hypothesis would require implausible assumptions of unreasonably high prior seasonal influenza attack rates, cross-protection against pH1N1 illness, and/or the effective block of that cross-protection by TIV (see Text S7 Appendix G).

We have also considered the possibility of a direct immune mechanism to explain our results. One such mechanism is based on the induction of low-affinity/non-neutralizing but cross-reactive antibodies by TIV to the major surface proteins. Such antibodies are typically identified within an “original antigenic sin” response [34]–[36], the relevance of which has long been debated for influenza, but for which Kim and colleagues have recently provided evidence in the mouse model [37]. In a related concept, antibody-dependent enhancement (ADE) of virus replication may occur when preexisting low levels of weakly heterotypic antibodies (cross-reactive but not cross-protective) bind to virus but instead of neutralizing the virus, form bridging complexes that facilitate enhanced uptake and increased virus production by the target cell [38]–[40]. Original antigenic sin and ADE of virus replication have been described for secondary heterotypic dengue infections, but also in vitro for other viruses such as respiratory syncytial virus, Ebola, HIV, etc. [38]–[40]. ADE has also been shown in vitro for influenza uptake by macrophages, although these are not generally considered the main target cell for influenza replication [41]–[44]. Interestingly, vaccine-potentiated pneumonia has been reported following heterologous swine influenza challenge in pigs, and enhanced pneumonia due to heterologous swine influenza has also been reported in piglets vaccinated in the presence of maternal antibody, with ADE invoked by authors to possibly explain these findings [45],[46]. In further support of a direct vaccine-induced effect to explain our findings, a recent pH1N1 challenge study of influenza-naïve ferrets has shown that, compared to a nonimmunized control group, prior receipt of 2008–09 seasonal vaccine (notably live attenuated vaccine) was associated with slight worsening of day 3 upper airway viral loads, clinical disease, and mortality, with ADE also invoked by authors as a possible explanation [47]. We did not find increased pH1N1 severity associated with seasonal vaccine in our epidemiologic studies; this observation may reflect intrinsically less virulent virus or, as described above, other intact viral clearance mechanisms [48].

In considering the ADE hypothesis, it should be recognized that this proposed mechanism has not been previously linked to epidemiologic observations for influenza in humans. ADE may require a precise balance of antigenic distance and cross-reactive versus neutralizing antibody to be manifest. The 2009 pandemic differs from other influenza epidemics or pandemics in that it has been caused by a novel virus distantly related to but nevertheless within the same HA/NA subtype as recently circulating H1N1 viruses. The role of antibody concentrations in ADE is under debate, so it is difficult to know whether our findings during spring–summer 2009 in Canada, if explained in this way, would have been observed during fall–winter 2009–10 with (or without) repeat administration of TIV, or whether seasonal vaccines may differ in the relative proportion of cross-reactive versus cross-protective antibody induced to pH1N1. In the end, our results may seem counterintuitive, but they cannot be dismissed on the basis that no biological mechanism can plausibly explain them. The mechanism may be a combination of the above or an as-yet unknown pathway. Further empiric evidence would be necessary to support a specific mechanism.

After the substantial autumn 2009 wave of pH1N1 and the mass pH1N1 vaccination campaign, seasonal influenza viruses remain a possible threat to consider for the rest of the 2009–10 winter. The complex benefit–risk analysis for receipt of 2009–10 TIV will ultimately depend upon the extent to which seasonal A/H1N1, A/H3N2, or B viruses contribute to TIV-preventable morbidity this season. It remains uncertain whether the influenza A subtype replacement observed with previous pandemics will also occur with this pandemic. Thus far (to 1 March 2010), seasonal strains have not comprised a substantial proportion of influenza detections in the northern hemisphere's 2009–10 season, with the exception of a recent increase in influenza B in China [49]. For the next season, WHO has recommended that pH1N1 be included in seasonal vaccine formulations, thereby providing direct pH1N1 protection and obviating the possible risk we identified in association with the seasonal vaccine in 2009 [50],[51]. The possible scientific implications of our findings, however, will remain important to consider over the long term. These include questions about influenza immunopathogenesis, the interaction between seasonal and novel pandemic strains, the complex immunoepidemiologic aspects of influenza prevention and control, and how best to assess these issues experimentally and epidemiologically.

In summary, we report findings from four epidemiologic studies in Canada showing that prior receipt of 2008–09 TIV was associated with increased risk of medically attended pH1N1 illness during the spring–summer 2009. Bias cannot be ruled out in observational studies, and therefore these findings cannot be considered conclusive. If these observations do reflect a real biological effect, however, they raise important questions that warrant further scientific investigation.

## Supporting Information

Text S1 Appendix A: Sentinel test-negative case-control study - Additional details.
(0.08 MB PDF)

Text S2 Appendix B: Quebec test-negative case-control study - Additional details.
(0.08 MB PDF)

Text S3 Appendix C: TIV coverage by age category estimated from Canadian Community Health Survey (2007–08 season).
(0.03 MB PDF)

Text S4 Appendix D: Quebec population case-control study - Additional details.
(0.05 MB PDF)

Text S5 Appendix E: Ontario test-negative case-control study - Additional details.
(0.05 MB PDF)

Text S6 Appendix F: Overall and antigenic region identity of 2009 pandemic H1N1 (pH1N1) proteins compared to representative recently circulating and vaccine strains of A/H1N1 and A/H3N2.
(0.03 MB PDF)

Text S7 Appendix G: Theoretical analysis of whether a 2-fold increased risk of pH1N1 associated with prior seasonal influenza vaccination could be explained by TIV effectively blocking the heterosubtypic cross-immunity provided by prior seasonal influenza infection.
(0.06 MB PDF)

## Abbreviations

ADE: antibody-dependent enhancement
BC: British Columbia
CCHS: Canadian Community Health Survey
CI,: confidence interval
HCW: health care worker
ILI: influenza-like illness
NA: neuraminidase
NP: nasopharyngeal
OR: odds ratio
pH1N1: pandemic influenza A (H1N1)
RR: risk ratio
SAR: secondary attack rate
SAVOIR: Studies of the Association of Vaccine on Influenza Risk
TIV: trivalent inactivated influenza vaccine
VE: vaccine effectiveness
WHO: World Health Organization
