# Supplementary material for: Association between the 2008–09 Seasonal Influenza Vaccine and Pandemic H1N1 Illness during Spring–Summer 2009: Four Observational Studies from Canada
Source: PLoS Med. 2010 Apr 6;7(4):e1000258. doi: 10.1371/journal.pmed.1000258 (PMC2850386; doi:10.1371/journal.pmed.1000258)
Supplement: Text S1 — Appendix A: Sentinel test-negative case-control study - Additional details. (0.08 MB PDF) [file pmed.1000258.s001.pdf]

Appendix A

Sentinel test-negative case-control study –
Additional details

Appendix A1. Additional methodological detail related to Canada's sentinel vaccine effectiveness monitoring system for 2008-09

The sentinel vaccine effectiveness study is based on a surveillance approach using a network of community-based practitioners provided with kits in order to submit respiratory specimens for influenza testing along with standardized epidemiologic information collected from eligible consenting patients. Both cases and controls are ambulatory patients consulting for influenza-like illness (ILI). Eligible participants in participating provinces (British Columbia (BC), Alberta, Ontario and Quebec) present within 7 days of ILI onset. ILI is defined as the acute onset of fever and cough and one or more of sore throat, myalgia, arthralgia, headache or prostration. Because trivalent split inactivated influenza vaccine (TIV) is only recommended for people ≥6 months of age and because this eligibility varied through the analysis period, infants <1 year of age were excluded from analyses. The National Advisory Committee on Immunization recommends two doses of TIV for previously unimmunized children < 9 years of age but one or two dose receipt was not verified. Children less than 3 years of age are recommended to receive 0.25mL per dose while all others receive 0.5mL per dose. TIV is administered intra-muscularly during the regular fall immunization campaign and immunization status is elicited from participants or parents at the time of medical visit for ILI, prior to specimen submission and testing by the designated provincial laboratory. Information on underlying health status is also collected prior to test result: in BC and Alberta, the questionnaire elicited whether the patient had a "vaccine-eligible" condition inclusive of heart, lung, renal, metabolic, blood or immune conditions; in Ontario and Quebec this question referred to "chronic" conditions inclusive of the same list. Submission of duplicate specimens is discouraged but not further assessed. Cases were participants who tested positive for influenza; controls were participants who tested negative for both influenza A and B. Per usual protocol, specimens positive for any influenza were excluded as controls (i.e. the specimens positive for seasonal influenza during the pH1N1 analysis period were excluded as controls as were those positive for pH1N1 when examining vaccine effectiveness against seasonal influenza during the same period). Because pH1N1 was not considered until after April 17, specimens with ILI onset in early April were not included in sentinel study analyses.

Testing for seasonal influenza in BC, Ontario and Quebec consisted of screening using a real-time reverse transcription PCR (RT-PCR) assay targeting the matrix (M) gene of influenza A virus and the hemagglutinin (HA) [1-3] or NS1 gene of influenza B virus (CDC real time RT-PCR protocol). Sub-typing for seasonal human influenza A (A/H1 or A/H3) was performed by real-time RT-PCR targeting the HA gene. In Alberta, all specimens were tested by Luminex RVP Assay [4] which detects seasonal influenza, pH1N1 [5] and influenza B viruses and directly subtypes seasonal influenza as A/H1 or A/H3.

Testing was appropriately modified in all provinces after April 17 to detect pH1N1. Owing to increased test volume, specimens were prospectively tested for influenza A alone by the screening real-time RT-PCR assay in BC and Ontario and were retrospectively tested for influenza B by Luminex RVP in BC and real-time RT-PCR in Ontario. In Quebec and Alberta, all specimens continued to be tested for both influenza A and B by screening RT-PCR or Luminex RVP respectively as per above. Influenza A positive specimens were subtyped by RT-PCR. The A/H1 and A/H3 subtypes were detected as described above. pH1N1 was detected using a conventional (end point) RT-PCR assay targeting the H1 gene using the forward primer CAGACACTGTAGACACAGTAC, and reverse primer CTAGTAGATGGATGGTGAATGC (NML Endpoint RT-PCR protocol distributed April 24, 2009). The 517 bp product was visualized by agarose gel electrophoresis or by real-time RT-PCR using hydrolysis probes on a Taqman platform to this region. Sequence analysis was also performed on a subset to confirm pH1N1 detection.

References:

1. Schweiger B, Zadow I, Heckler R, Timm H, Pauli G. (2000) Application of a fluorogenic PCR assay for typing and subtyping of influenza viruses in respiratory samples. J Clin Microbiol 38:1552-1558.
2. van Elden LJ, Nijhuis M, Schipper P, Schuurman R, van Loon AM. (2001) Simultaneous detection of influenza viruses A and B using real-time quantitative PCR. J Clin Microbiol 39:196-200.
3. Fouchier, R.A, Bestebroer, TM, Herfst S, Van Der Kemp L, Rimmelzwaan GF et al. (2000) Detection of influenza A viruses from different species by PCR amplification of conserved sequences in the matrix gene. J. Clin. Microbiol. 38:4096–4101.
4. Mahony JS, Chong F, Merante S, Yaghoubian T, Sinha C, et al. (2007) Development of a respiratory virus panel test for detection of twenty human respiratory viruses by use of multiplex PCR and a fluid microbead-based assay. J. Clin. Microbiol. 45:2965-2970.
5. Mahony JB, Hatchette T, Ojkic D, Drews SJ, Gubbay J, et al. (2009) Multiplex PCR tests sentinel the appearance of pandemic influenza viruses including H1N1 swine influenza. J Clin Virol 45:200-202.

Appendix A2. Sentinel test-negative case-control study participant inclusion and exclusion – seasonal influenza and pH1N1 analysis periods

Exclusion Criteria	Seasonal November 1, 2008 – March 31, 2009		pH1N1 April 17, 2009 – July 22, 2009	
	N excluded	N included	N excluded	N included
Submitted from consenting patients within study period		1775		936
Patient did not meet influenza-like illness (ILI) case definition or unknown	71	1704	62	874
Vaccine status unknown	32	1672	57	817
Specimen collected > 7 days after ILI onset or interval unknown	77	1595	52	765
Vaccine-eligible (chronic) conditions unknown	36	1559	39	726
Age unknown	3	1556	1	725
Age < 1 year	12	1544	8	717
Influenza RT-PCR results unavailable or inconclusive on typing	15	1529	6	711
Specimens positive for seasonal influenza or with subtype unknown or pending (pH1N1 analysis period)	N/A	1529	31	680
Contributed to analysis		1529		680

pH1N1= pandemic influenza A (H1N1); ILI= influenza-like illness defined as acute onset of fever and cough and one or more of sore throat, arthralgia, myalgia, headache or prostration; RT-PCR=reverse-transcription polymerase chain reaction; N/A=Not applicable.

Appendix A3a: Laboratory profile of included sentinel specimens with dates of ILI onset November 1, 2008 - March 31, 2009

Specimens tested	Alberta N=521	BC N=380	Ontario N=292	Quebec N=336	Total N=1529
	n (%)	n (%)	n (%)	n (%)	n (%)
**Influenza negative**	375 (72%)	189 (50%)	166 (57%)	127 (38%)	857 (56%)
**Influenza positive**	146 (28%)	191 (50%)	126 (43%)	209 (62%)	672 (44%)
**A positive**	94 (64%)	122 (64%)	71 (56%)	136 (65%)	423 (63%)
**B positive**	52 (36%)	69 (36%)	55 (44%)	73 (35%)	249 (37%)
**Influenza A positive**					
A/H1	43 (46%)	38 (31%)	36 (51%)	43 (32%)	160 (38%)
A/H3	50 (53%)	84 (69%)	29 (41%)	90 (66%)	253 (60%)
Subtype unknown	1 (1%)	0 (0%)	6 (9%)	3 (2%)	10 (2%)
pH1N1	0 (0%)	0 (0%)	0 (0%)	0 (0%)	0 (0%)

Appendix A3b: Laboratory profile of included sentinel specimens with dates of ILI onset April 17, 2008-July 22, 2009

Specimens tested	Alberta N=202	BC N=197	Ontario N=140	Quebec N=172	Total N=711
	n (%)	n (%)	n (%)	n (%)	n (%)
**Influenza negative**	167 (83%)	166 (84%)	102 (73%)	101 (59%)	536 (75%)
**Influenza positive**	35 (17%)	31 (16%)	38 (27%)	71 (41%)	175 (25%)
**A positive**	31 (89%)	28 (90%)	38 (100%)	67 (94%)	164 (94%)
**B positive**	4 (11%)	3 (10%)	0 (0%)	4 (6%)	11 (6%)
**Influenza A positive**					
A/H1	1 (3%)	1 (4%)	1 (3%)	1 (2%)	4 (2%)
A/H3	2 (7%)	8 (29%)	2 (5%)	2 (3%)	14 (9%)
Subtype unknown	1 (3%)	0 (0%)	1 (3%)	0 (0%)	2 (1%)
pH1N1	27 (87%)	19 (68%)	34 (90%)	64 (96%)	144 (88%)

pH1N1= pandemic influenza A (H1N1)
ILI=influenza-like illness

Appendix A4. Influenza detection by subtype and week (based on date of ILI onset), 2008-09: British Columbia, Alberta, Ontario and Quebec, combined among sentinel study participants meeting inclusion/exclusion criteria (n=2324)*

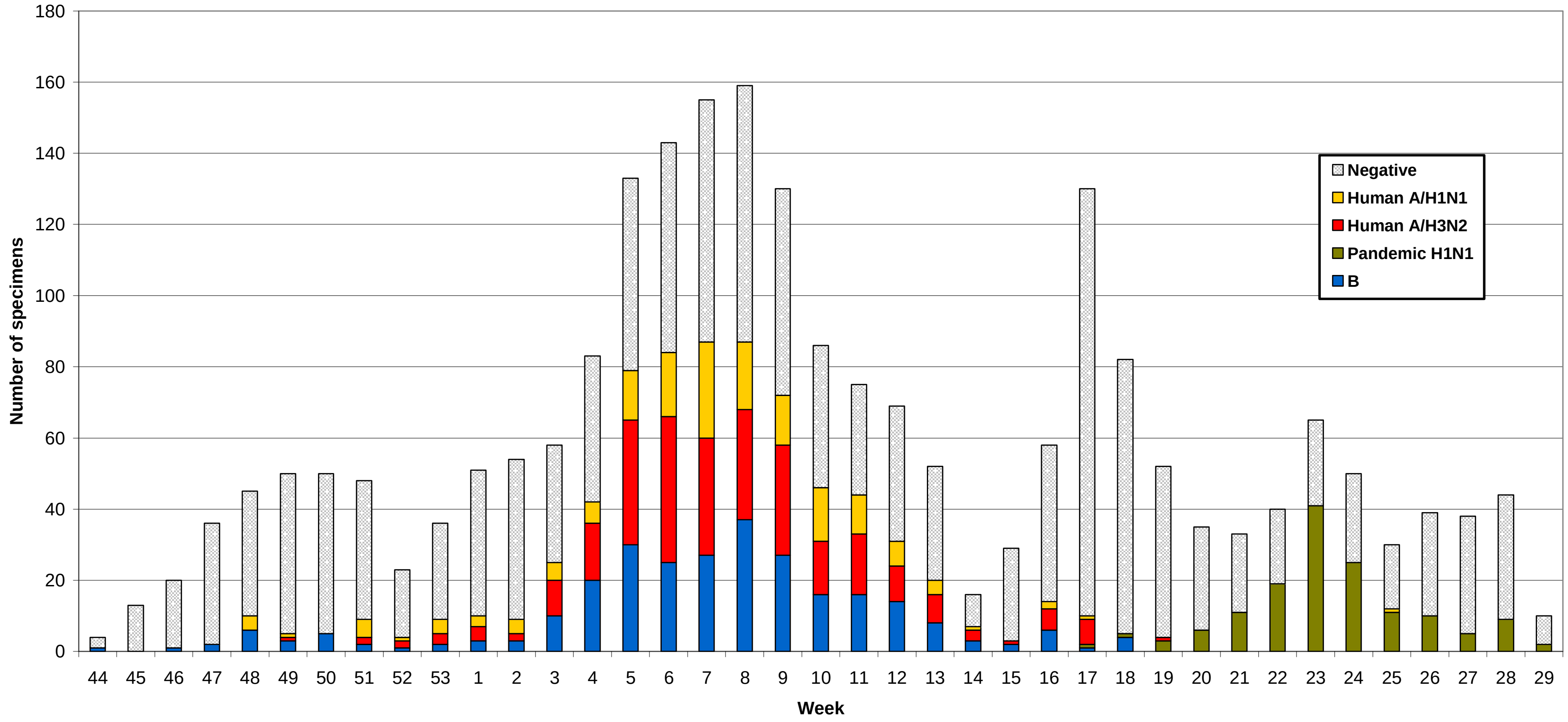

*Twelve specimens of unknown subtype not represented in this figure.
ILI=influenza-like illness

NOTE: Week 44 = 26 October, 2008 – 1 November, 2008; Week 29 = 19 July, 2009 – 25 July, 2009

NOTE: 1 November, 2008 in week 44; 31 March, 2009 in week 13; 17 April, 2009 in week 15; 17 May, 2009 in week 20; 22 July, 2009 in week 29.

Table A5a: Sentinel test-negative case-control study: participant profile, seasonal influenza

	Seasonal influenza test-positive cases (N=672) n/N (%)								Seasonal influenza test-negative controls (N=857) n/N (%)								ALL
	**1-8 years**	**9-19 years**	**20-49 years**	**50-64 years**	**≥ 65 years**	**< 50 years**	**≥ 50 years**	**Overall**	**1-8 years**	**9-19 years**	**20-49 years**	**50-64 years**	**≥ 65 years**	**< 50 years**	**≥ 50 years**	**Overall**	
**N**	**100 n (%)**	**176 n (%)**	**311 n (%)**	**69 n (%)**	**16 n (%)**	**587 n (%)**	**85 n (%)**	**672 n (%)**	**93 n (%)**	**123 n (%)**	**384 n (%)**	**172 n (%)**	**85 n (%)**	**600 n (%)**	**257 n (%)**	**857 n (%)**	**1529 n (%)**
**2008-09 TIV Receipt**	14 (14)	14 (8)	39 (12)	17 (25)	13 (81)	67 (11)	30 (35)	97 (14)	20 (22)	23 (19)	86 (22)	84 (49)	74 (87)	129 (22)	158 (61)	287 (33)	384 (25)
**Sex Female**	48 (48)	93 (53)	182 (58)	38 (55)	10 (63)	323 (55)	48 (56)	371 (55)	45 (48)	71 (58)	224 (58)	103 (60)	60 (71)	340 (57)	163 (63)	503 (59)	874 (57)
**Interval between ILI onset and specimen collection**																	
≤ 4 days	80 (80)	153 (87)	266 (86)	58 (84)	12 (75)	499 (85)	70 (82)	569 (85)	77 (83)	99 (80)	297 (77)	119 (69)	59 (69)	473 (79)	178 (69)	651 (76)	1220 (80)
5-7 days	20 (20)	23 (13)	45 (14)	11 (16)	4 (25)	88 (11)	15 (18)	103 (15)	16 (17)	24 (19)	87 (23)	53 (31)	26 (31)	127 (21)	79 (31)	206 (24)	309 (20)
Median (range)	3 (0-7)	3 (0-7)	3 (0-7)	3 (0-6)	3 (1-6)	3 (0-7)	3 (0-6)	3 (0-7)	3 (0-7)	3 (0-7)	3 (0-7)	3 (0-7)	3 (0-7)	3 (0-7)	3 (0-7)	3 (0-7)	3 (0-7)
**With Chronic conditions**																	
**N**	6 (6)	14 (8)	41 (13)	17 (25)	11 (69)	61 (10)	28 (33)	89 (13)	13 (14)	12 (10)	53 (14)	64 (37)	57 (67)	78 (13)	121 (47)	199 (23)	288 (19)
Vaccinated 2008-09	2 (33)	5 (36)	16 (39)	6 (35)	8 (73)	23 (38)	14 (50)	37 (42)	5 (38)	7 (58)	27 (51)	44 (69)	50 (88)	39 (50)	94 (78)	133 (67)	170 (59)
**Without chronic conditions**																	
**N**	94 (94)	162 (92)	270 (87)	52 (75)	5 (31)	526 (90)	57 (67)	583 (87)	80 (86)	111 (90)	331 (86)	108 (63)	28 (33)	522 (87)	136 (53)	658 (77)	1241 (81)
Vaccinated 2008-09	12 (13)	9 (6)	23 (8)	11 (21)	5 (100)	44 (8)	16 (28)	60 (10)	15 (19)	16 (14)	59 (18)	40 (37)	24 (86)	90 (17)	64 (47)	154 (23)	214 (17)
**Province**																	
Alberta	19 (19)	36 (20)	77 (25)	13 (19)	1 (6)	132 (22)	14 (16)	146 (22)	35 (38)	61 (50)	176 (46)	74 (43)	29 (34)	272 (45)	103 (40)	375 (44)	521 (34)
BC	23 (23)	64 (36)	91 (29)	8 (12)	5 (31)	178 (30)	13 (15)	191 (28)	11 (12)	27 (22)	100 (26)	29 (17)	22 (26)	138 (23)	51 (20)	189 (22)	380 (25)
Ontario	18 (18)	27 (15)	60 (19)	15 (22)	6 (38)	105 (18)	21 (25)	126 (19)	21 (23)	25 (20)	58 (15)	42 (24)	20 (23)	104 (17)	62 (24)	166 (19)	292 (19)
Quebec	40 (40)	49 (28)	83 (27)	33 (48)	4 (25)	172 (29)	37 (43)	209 (31)	26 (28)	10 (8)	50 (13)	27 (16)	14 (16)	86 (14)	41 (16)	127 (15)	336 (22)
**Chronic conditions by province**																	
Alberta	0	5 (14)	12 (16)	2 (15)	0	17 (13)	2 (14)	19 (13)	6 (17)	10 (16)	31 (18)	29 (39)	23 (79)	47 (17)	52 (50)	99 (26)	118 (23)
BC	4 (17)	4 (6)	13 (14)	1 (12)	5 (100)	21 (12)	6 (46)	27 (14)	2 (18)	0	13 (13)	11 (38)	14 (64)	15 (11)	25 (49)	40 (21)	67 (18)
Ontario	1 (6)	2 (7)	11 (18)	3 (20)	3 (50)	14 (13)	6 (29)	20 (16)	1 (5)	2 (8)	3 (5)	15 (36)	12 (60)	6 (6)	27 (44)	33 (20)	53 (18)
Quebec	1 (3)	3 (6)	5 (6)	11 (33)	3 (75)	9 (5)	14 (38)	23 (11)	4 (15)	0	6 (12)	9 (33)	8 (57)	10 (12)	17 (41)	27 (21)	50 (15)
**2008-09 TIV immunized by province**																	
Alberta	2 (10)	4 (11)	7 (9)	1 (8)	1 (100)	13 (10)	2 (14)	15 (10)	8 (23)	11 (18)	37 (21)	34 (46)	23 (79)	56 (21)	57 (55)	113 (30)	128 (25)
BC	1 (4)	4 (6)	11 (12)	0	4 (80)	16 (9)	4 (31)	20 (10)	0 (0)	4 (15)	18 (18)	12 (41)	21 (95)	22 (16)	33 (65)	55 (29)	75 (20)
Ontario	3 (17)	5 (18)	13 (22)	6 (40)	5 (83)	21 (20)	11 (52)	32 (25)	6 (29)	7 (28)	21 (36)	24 (57)	17 (85)	34 (33)	41 (66)	75 (45)	107 (37)
Quebec	8 (20)	1 (2)	8 (10)	10 (30)	3 (75)	17 (10)	13 (35)	30 (14)	6 (23)	1 (10)	10 (20)	14 (52)	13 (93)	17 (20)	27 (66)	44 (35)	74 (22)
**2007-08 TIV immunized by province***																	
Alberta	3 (16)	5 (14)	16 (22)	3 (23)	1 (100)	24 (19)	4 (29)	28 (20)	6 (17)	14 (23)	40 (23)	36 (49)	25 (86)	60 (22)	61 (60)	121 (33)	149 (29)
Ontario	5 (29)	8 (32)	21 (36)	7 (50)	6 (0)	34 (34)	13 (65)	47 (39)	7 (35)	8 (33)	24 (44)	25 (61)	16 (84)	39 (40)	41 (68)	80 (51)	127 (46)
Quebec	8 (21)	0	13 (16)	9 (28)	3 (75)	21 (13)	12 (33)	33 (16)	7 (29)	0	7 (15)	14 (52)	12 (92)	14 (17)	26 (65)	40 (33)	73 (22)

ILI=influenza-like illness; TIV=trivalent inactivated influenza vaccine; BC=British Columbia; *BC did not collect information on 2007-08 vaccine status. 2007-08 percentages derived based on participants for whom vaccine status known. Denominators do not include those with missing information (never exceeding 4 missing counts for any age category).

Table A5b: Sentinel test-negative case-control study: participant profile, pH1N1 influenza

	pH1N1 test-positive cases (N=144) n/N (%)								pH1N1 test-negative controls (N=536)* n/N (%)								ALL
	1-8 years	9-19 years	20-49 years	50-64 years	≥ 65 years	< 50 years	≥ 50 years	Overall	1-8 years	9-19 years	20-49 years	50-64 years	≥ 65 years	< 50 years	≥ 50 years	Overall	
**N**	**13**	**59**	**59**	**10**	**3**	**131**	**13**	**144**	**41**	**66**	**272**	**109**	**48**	**379**	**157**	**536**	**680**
	**n (%)**	**n (%)**	**n (%)**	**n (%)**	**n (%)**	**n (%)**	**n (%)**	**n (%)**	**n (%)**	**n (%)**	**n (%)**	**n (%)**	**n (%)**	**n (%)**	**n (%)**	**n (%)**	**n (%)**
**2008-09 TIV* Receipt**	5 (38)	13 (22)	22 (37)	2 (20)	3 (100)	40 (30)	5 (38)	45 (31)	4 (10)	6 (9)	63 (23)	60 (55)	33 (69)	73 (19)	93 (59)	166 (31)	211 (31)
**Sex Female**	7 (54)	28 (47)	37 (63)	5 (50)	2 (67)	72 (55)	7 (54)	79 (55)	14 (34)	34 (51)	184 (68)	74 (68)	26 (55)	232 (61)	100 (64)	332 (62)	411 (60)
**Interval between ILI onset and specimen collection**																	
≤ 4 days	10 (77)	54 (91)	52 (88)	10 (100)	2 (67)	116 (89)	12 (92)	128 (89)	34 (83)	56 (85)	222 (82)	71 (65)	35 (73)	312 (82)	106 (67)	418 (78)	546 (80)
5-7 days	3 (23)	5 (8)	7 (12)	0 (0)	1 (33)	15 (11)	1 (8)	16 (11)	7 (17)	10 (15)	50 (18)	38 (35)	13 (27)	67 (18)	51 (32)	118 (22)	134 (20)
Median (range)	2 (0-7)	2 (0-5)	2 (0-7)	2 (1-3)	2 (1-7)	2 (0-7)	2 (1-7)	2 (0-7)	2 (0-7)	3 (0-7)	3 (0-7)	3 (0-7)	3 (0-7)	3 (0-7)	3 (0-7)	3 (0-7)	3 (0-7)
**With Chronic conditions**																	
**N**	4 (31)	4 (7)	9 (15)	3 (30)	3 (100)	17 (13)	6 (46)	23 (16)	1 (2)	1 (1)	39 (14)	34 (31)	27 (56)	41 (11)	61 (39)	102 (19)	125 (18)
Vaccinated 2008-09	2 (50)	3 (75)	5 (56)	1 (33)	3 (100)	10 (59)	4 (67)	14 (61)	1 (100)	1 (100)	19 (49)	20 (74)	20 (74)	21 (51)	40 (66)	61 (60)	75 (60)
**Without chronic conditions**																	
**N**	9 (69)	55 (93)	50 (85)	7 (70)	0	114 (87)	7 (54)	121 (84)	40 (98)	65 (98)	233 (86)	75 (69)	21 (44)	338 (89)	96 (61)	434 (81)	555 (82)
Vaccinated 2008-09	3 (33)	10 (18)	17 (34)	1 (14)	0	30 (26)	1 (14)	31 (26)	3 (8)	5 (8)	44 (19)	40 (53)	13 (62)	52 (15)	53 (55)	105 (24)	136 (24)
**Province**																	
Alberta	0	9 (15)	13 (22)	3 (30)	2 (67)	22 (17)	5 (38)	27 (19)	10 (24)	23 (35)	87 (32)	33 (30)	14 (29)	120 (32)	47 (30)	167 (31)	194 (29)
BC	1 (8)	6 (10)	10 (17)	2 (20)	0	17 (13)	2 (15)	19 (13)	10 (24)	16 (24)	89 (33)	34 (31)	17 (35)	115 (30)	51 (32)	166 (31)	185 (27)
Ontario	4 (31)	16 (27)	13 (22)	1 (10)	0	33 (25)	1 (8)	34 (24)	11 (27)	11 (17)	46 (17)	22 (20)	12 (25)	68 (18)	34 (22)	102 (19)	136 (20)
Quebec	8 (61)	28 (47)	23 (39)	4 (40)	1 (33)	59 (45)	5 (38)	64 (44)	10 (24)	16 (24)	50 (18)	20 (18)	5 (10)	76 (20)	25 (16)	101 (19)	165 (24)
**Chronic conditions by province**																	
Alberta	0	0 (0)	3 (23)	1 (33)	2 (100)	3 (14)	3 (60)	6 (22)	0	1 (4)	12 (14)	14 (42)	7 (50)	13 (11)	21 (45)	34 (20)	39 (20)
BC	1 (100)	0 (0)	3 (30)	2 (100)	0	4 (24)	2 (100)	6 (32)	0	0	15 (17)	11 (32)	13 (76)	15 (13)	24 (47)	39 (23)	45 (24)
Ontario	0	2 (12)	2 (15)	0	0	4 (12)	0 (0)	4 (12)	0	0	3 (6)	3 (14)	4 (33)	3 (4)	7 (21)	10 (10)	14 (10)
Quebec	3 (37)	2 (7)	1 (4)	0	1 (100)	6 (10)	1 (20)	7 (11)	1 (10)	0	9 (18)	6 (30)	3 (60)	10 (13)	9 (36)	19 (19)	26 (16)
**2008-09 TIV immunized by province**																	
Alberta	0	1 (11)	5 (38)	1 (33)	2 (100)	6 (27)	3 (60)	9 (33)	0	3 (13)	27 (31)	18 (55)	8 (57)	30 (25)	26 (55)	56 (34)	65 (34)
BC	0	0	3 (30)	1 (50)	0	3 (18)	1 (50)	4 (21)	0	0 (0)	18 (20)	18 (53)	10 (59)	18 (16)	28 (55)	46 (28)	50 (27)
Ontario	2 (50)	5 (31)	4 (31)	0 (0)	0	11 (33)	0	11 (32)	2 (18)	2 (18)	10 (22)	13 (59)	10 (83)	14 (21)	23 (68)	37 (36)	48 (35)
Quebec	3 (37)	7 (25)	10 (43)	0 (0)	1 (100)	20 (34)	1 (20)	21 (33)	2 (20)	1 (6)	8 (16)	11 (55)	5 (100)	11 (14)	16 (64)	27 (27)	48 (29)
**2007-08 TIV immunized by province***																	
Alberta	0	1 (11)	4 (31)	2 (67)	1 (50)	5 (23)	3 (60)	8 (30)	1 (10)	3 (15)	24 (29)	14 (44)	10 (71)	28 (25)	24 (52)	52 (33)	52 (28%)
Ontario	1 (33)	6 (40)	7 (70)	0	0	14 (50)	0	14 (48)	1 (9)	7 (64)	14 (31)	11 (52)	8 (67)	22 (33)	19 (58)	41 (41)	41 (32%)
Quebec	3 (38)	6 (23)	8 (40)	0	1 (100)	17 (31)	1 (20)	18 (31)	3 (33)	2 (13)	9 (18)	9 (50)	5 (100)	14 (19)	14 (61)	28 (29)	28 (18)

pH1N1= pandemic influenza A (H1N1); ILI=influenza-like illness; TIV=trivalent inactivated influenza vaccine; BC=British Columbia; *BC did not collect information on 2007-08 vaccine status. 2007-08 percentages derived based on participants for whom vaccine status known. Denominators do not include those with missing information (never exceeding 3 missing counts for any age category).
