# Supplementary material for: Association between the 2008–09 Seasonal Influenza Vaccine and Pandemic H1N1 Illness during Spring–Summer 2009: Four Observational Studies from Canada
Source: PLoS Med. 2010 Apr 6;7(4):e1000258. doi: 10.1371/journal.pmed.1000258 (PMC2850386; doi:10.1371/journal.pmed.1000258)
Supplement: Text S2 — Appendix B: Quebec test-negative case-control study - Additional details. (0.08 MB PDF) [file pmed.1000258.s002.pdf]

Appendix B

Quebec test-negative case-control study –
Additional details

Appendix B1a. Quebec pH1N1 case-control study: test-negative approach

Initially, the Quebec pandemic influenza A/H1N1 (pH1N1) case-control study planned to use pH1N1 test-negative community controls. There were 1442 patients identified who were test-negative for pH1N1: 808 aged <50 years and 634 who were ≥50 years. The planned sample size was 300 test-negatives age < 50 years and 150 test-negatives age ≥50 years. A random sample of twice the target number of patients was drawn (600 + 300 = 900) and ultimately 475 (303 and 172, respectively) responded (Appendix B1b).

Among the 475 pH1N1 test-negative controls reached, 59 were excluded at the analysis stage (Appendix B1b). Among the 416 remaining pH1N1 test-negative participants, 50% (206) had a chronic condition - higher than community cases (96; 25%) and far exceeding estimates from the general population. Among pH1N1 test-negative controls, 13% (54/416) were tested without symptoms (Appendix B1c). Whereas 68% (261/383) of community cases met the influenza-like illness (ILI) case definition, only 127 (30%) of pH1N1 test-negative controls met this criterion and 15% (64/416) had their specimen collected >7 days after illness onset, reducing the likelihood of virus detection. Among the 224 pH1N1 test-negative controls aged 20-59 years, 35% (80) were health care workers (HCWs) compared to 6% (7/127) of hospitalized cases and 33% (64/195) of community cases. Finally, receipt of trivalent inactivated influenza vaccine (TIV) by test-negative controls far exceeded rates in the general population of Quebec (Appendix B1c and see also Appendix C).

The crude odds ratios (ORs) for pH1N1 associated with 2008-2009 TIV were 0.76 [95%CI 0.57-1.01] and 1.00 [95%CI 0.73-1.35] when comparing community and hospitalized cases respectively to test-negative controls (Appendix B1d). After adjustment for age, chronic conditions, sex and health care worker (HCW) status the ORs were 1.12 [0.80-1.57] and 1.35 [0.94-1.94], respectively. After we standardized for clinical presentation by restricting to participants that met the ILI case definition, adjusted ORs were 1.38 [0.80-2.40] and 1.52 [0.85-2.73]. After restricting to patients meeting ILI and from whom the specimen was collected within 7days of illness onset, adjusted ORs were almost unchanged at 1.42 [0.76-2.68] and 1.50 [0.73-2.95] (Appendix B1d/B1e). When HCWs were excluded, and analysis was restricted to patients with ILI and specimen collection within 7 days of illness onset, the adjusted ORs were 1.66 [0.75-3.67] and 1.96 [0.91-4.24].

After May 15th in Quebec, patient testing for pH1N1 was limited outside the sentinel system to patients with severe illness possibly requiring hospitalization. This resulted in a skewed sampling frame for selection of controls based on test-negative results among general provincial laboratory specimens. Several aspects of the pH1N1 test-negative participant profile indicated this group of controls was inadequate: the high proportion with chronic conditions as compared to cases; the high proportion immunized as compared to the general population; the small proportion with ILI; and the substantial proportion tested >7 days after illness onset (see Table 4 and Appendices B1c, C and D2). We therefore resorted to the conventional case-control approach of selecting controls directly from the community. The Quebec experience with the test-negative case-control approach based on specimens drawn from general laboratory submissions during a period when restrictions on testing were applied is an important reminder that results from such design, as with all observational studies, should be carefully scrutinized for evidence of possible selection bias operating in either direction.

Appendix B1b: Quebec pH1N1 test-negative case-control approach – exclusion criteria and participation by study group

	Community pH1N1 cases	Hospitalized pH1N1 cases	pH1N1 negative controls
Reported cases	993	415	900
Phone number unknown	343	48	197
No response/not reached	176	25	181
Phone contact made	441	342	522
Refusal	11	4	36
Not able to speak English/French	1	1	11
Before exclusions	395 (40%*)	337 (81%*)	475 (91%*)
Exclusions	11	67	59
< 6 month (Dec 1st 2008)	6	19	43
Hospitalized for reason other than H1N1	NA	15	NA
Unknown vaccination status	5	33	16
Final participants	384	270	416
Meeting ILI criteria	261 (68%†)	159 (59%†)	127 (30%†)
Specimen taken ≤7 days after illness onset	355 (92%†)	237 (88%†)	297 (71%†)
Meeting ILI and had specimen taken ≤7 days after illness onset	237 (62%†)	144 (53%†)	95 (23%†)

pH1N1= pandemic influenza A (H1N1)
* % calculated over reported cases
† % calculated over final participants
ILI=influenza-like illness defined as fever and cough and one or more of sore throat, myalgia, arthralgia, headache or prostration

Appendix B1c: Quebec pH1N1 test-negative case control: characteristics of pH1N1 negative controls

	6-59 months	5-19 years	20-34 years	35-49 years	50-59 years	≥60 years	Total
Before exclusion	93	45	84	81	63	109	475
Exclusions	46	6	1	2	1	3	59
**Final participants (N)**	**47**	**39**	**83**	**79**	**62**	**106**	**416**
	n (%)	n (%)	n (%)	n (%)	n (%)	n (%)	
Received 2008-09 TIV	17 (36)	12 (31)	33 (40)	28 (35)	28 (45)	66 (62)	184
Sex female	16 (34)	17 (43)	60 (72)	47 (59)	36 (58)	49 (46)	225
Health care workers	0 (0)	1 (3)	36 (43)	25 (32)	19 (31)	3 (3)	84
**Symptoms**							
None	6 (13)	3 (8)	8 (10)	3 (4)	9 (14)	25 (24)	54
≥1 influenza symptom	41 (87)	36 (92)	75 (90)	76 (96)	53 (85)	80 (75)	361
Fever	37 (79)	29 (74)	55 (66)	61 (77)	37 (60)	52 (49)	271
Fever and cough	14 (30)	19 (49)	31 (37)	36 (46)	24 (39)	30 (28)	154
ILI (Fever and cough + 1 other symptom)	9 (19)	12 (31)	25 (30)	35 (44)	21 (34)	25 (24)	127
**Number of days between disease onset and specimen collection**							
0-3	25 (53)	18 (46)	48 (59)	37 (47)	26 (42)	41 (39)	195
4-7	10 (21)	6 (15)	17 (20)	21 (26)	15 (24)	15 (14)	84
> 7 days	4 (8)	9 (23)	8 (10)	17 (21)	10 (16)	16 (15)	64
**Previous Influenza Vaccination (TIV)**							
No chronic condition	32	23	62	44	20	29	210
Vaccinated in 2008-2009	9 (28)	8 (35)	25 (40)	18 (41)	9 (45)	12 (41)	81
With chronic condition	15	16	21	35	42	77	206
Vaccinated in 2008-2009	8 (53)	4 (25)	8 (38)	10 (28)	19 (45)	54 (70)	103
**Participants with ILI and specimen collection ≤7 days after disease onset**	6(13)	7 (18)	21 (25)	25 (32)	17 (27)	16 (15)	92

pH1N1= pandemic influenza A (H1N1)
ILI=influenza-like illness; TIV=trivalent inactivated influenza vaccine
NOTE: where percentages do not sum 100% this is owing to missing data

Appendix B1d: Quebec pH1N1 test-negative case-control study: OR (95% CI) for pH1N1 associated with TIV

	Community pH1N1 cases *vs.* pH1N1 negative controls	Hospitalized pH1N1 cases *vs.* pH1N1 negative controls
**All patients**	N=384 vs. 416	N=270 vs. 416
OR crude	0.76 (0.57-1.01)	1.00 (0.73-1.35)
Adjusted for age, sex chronic condition, HCW	1.12 (0.80-1.57)	1.35 (0.94-1.94)
**Patients excluding HCW**	N=316 vs. 332	N=262 vs. 332
OR crude	0.66 (0.48-0.92)	1.12 (0.81-1.56)
Adjusted for age, sex chronic condition	1.12 (0.75-1.67)	1.42 (0.97-2.06)
**Patients with ILI**	N=264 vs. 127	N= 159 vs. 127
OR crude	0.97 (0.63-1.50)	1.08 (0.67-1.74)
Adjusted for age, sex, chronic condition, HCW	1.38 (0.80-2.40)	1.52 (0.85-2.73)
**Patients with ILI and delay between onset of symptoms and specimen collection ≤7 days**	N=236 vs. 92	N= 134 vs. 92
OR crude	1.04 (0.63-1.71)	1.13 (0.66-1.95)
Adjusted for age, sex chronic condition, HCW	1.42 (0.76-2.68)	1.50 (0.73-2.95)
**Patients with ILI and delay between onset of symptoms and specimen collection ≤7 days, excluding HCW**	N=188 vs. 71	N= 129 vs. 71
OR crude	0.95 (0.53-1.71)	1.45 (0.80-2.67)
Adjusted for age, sex, chronic condition	1.66 (0.75-3.67)	1.96 (0.91-4.24)

pH1N1= pandemic influenza A (H1N1)
ILI=influenza-like illness; TIV=trivalent inactivated influenza vaccine

Appendix B1e: Quebec pH1N1 test-negative case-control study: OR (95% CI) for pH1N1 associated with TIV in patients with ILI and with delay between onset of symptoms and specimen collection ≤7 days after illness onset

	Community pH1N1 cases *vs.* pH1N1 negative controls	Hospitalized pH1N1 cases *vs.* pH1N1 negative controls
**OR for previous 2008-2009 TIV**	N=236 vs. 92	N=134 vs. 92
**Crude**	1.04 (0.63-1.71)	1.13 (0.65-1.95)
**Adjusted for**:		
Age (ref: 20-34 years)	1.40 (0.80-2.43)	1.47 (0.80-2.70)
Chronic condition	1.21 (0.72-2.03)	1.00 (0.57-1.76)
Sex female	1.07 (0.65-1.76)	1.16 (0.67-2.01)
Region	0.98 (0.59-1.65)	1.04 (0.60-1.82)
Heath care workers	1.08 (0.64-1.82)	1.32 (0.74-2.36)
Age + chronic condition	1.50 (0.84-2.64)	1.31 (0.70-2.44)
Age + chronic condition + sex	1.50 (0.84-2.65)	1.33 (0.71-2.48)
Age + chronic condition + sex + region	1.44 (0.79-2.62)	1.14 (0.60-2.18)
Age + chronic condition + sex + region +heath care workers (HCW)	1.42 (0.76-2.68)	1.50 (0.73-2.95)
**Adjusted* OR for other seasonal influenza immunization**		
Immunized in 2007-2008	N=232 vs. 88<br>1.09 (0.58-2.03)	N=129 vs. 88<br>1.13 (0.54-2.38)
Immunized in 2007-08 and 2008-09	1.17 (0.61-2.24)	1.43 (0.66-3.09)
≥ once in last 5 years vs. never	N=234 vs. 89<br>1.18 (0.64-2.18)	N=122 vs. 89<br>1.20 (0.60-2.38)
5 times in last 5 years vs. never	N=149 vs. 58<br>1.21 (0.51-2.85)	N=81 vs. 58<br>1.77 (0.66-4.76)
**Adjusted OR for previous 2008-2009 TIV by strata**		
**Chronic condition** †		
None	N=174 vs. 45<br>1.86 (0.78-4.44)	N=48 vs. 45<br>2.52 (0.55-11.61)
With chronic condition	N =62 vs. 47<br>0.93 (0.32-2.65)	N=86 vs. 47<br>1.88 (0.73-4.78)
**Age stratified at 50 years**		
< 50 years ‡	N=200 vs. 59<br>1.95 (0.92-4.16)	N=96 vs. 59<br>1.62 (0.63-4.13)
≥50 years §	N=36 vs. 33<br>0.93 (0.23-2.98)	N=38 vs. 33<br>1.54 (0.46-5.13)

pH1N1= pandemic influenza A (H1N1)
ILI=influenza-like illness; TIV=trivalent inactivated influenza vaccine
* Adjusted for Age + chronic condition + sex + region +health care workers (HCW)
† Adjusted for Age + sex + region + HCW
‡ Adjusted for age (<10, 10-19 yrs and 20-45 (ref)) chronic condition + sex + region +HCW
§ Adjusted for age (50-59 (ref) and ≥60 yrs) + chronic condition + sex + region +HCW
