# Supplementary material for: Association between the 2008–09 Seasonal Influenza Vaccine and Pandemic H1N1 Illness during Spring–Summer 2009: Four Observational Studies from Canada
Source: PLoS Med. 2010 Apr 6;7(4):e1000258. doi: 10.1371/journal.pmed.1000258 (PMC2850386; doi:10.1371/journal.pmed.1000258)
Supplement: Text S3 — Appendix C: TIV coverage by age category estimated from Canadian Community Health Survey (2007–08 season). (0.03 MB PDF) [file pmed.1000258.s003.pdf]

Appendix C

Trivalent inactivated influenza vaccine (TIV) coverage by age category estimated from the Canadian Community Health survey (2007-08 season)

Appendix C: Trivalent inactivated influenza vaccine (TIV) coverage by age category estimated from the Canadian Community Health Survey (2007-08 season)*

	TIV coverage (95% CI) by age category					
	12-19 years	20-34 years	35-44 years	45-64 years	≥ 65 years	≥ 12 years
BC	24% (21-27)	17% (15-19)	22% (20-25)	32% (30-34)	64% (62-67)	31% (30-32)
Alberta	21% (18-24)	18% (16-21)	21% (18-23)	28% (26-30)	65% (62-68)	27% (26-28)
Ontario	30% (28-34)	20% (19-22)	27% (25-28)	41% (39-42)	73% (72-75)	37% (36-37)
Quebec	10% (9-12)	13% (11-14)	19% (17-21)	27% (25-29)	63% (61-65)	26% (25-27)
Canada	23% (22-24)	17% (17-18)	23% (22-24)	34% (33-35)	68% (67-69)	32% (31-32)

*Statistics Canada (2009) Influenza Immunization Coverage by Province and age 2007-08. In: Table 105-0502. Health indicator profile, annual estimates, by age group and sex, Canada, provinces, territories, health regions (2007 boundaries) and peer groups, occasional (1209600 series). Available at: http://cansim2.statcan.gc.ca/cgi-win/cnsmcgi.pgm?Lang=E&RootDir=CII/&Array_Pick=1&ArrayId=105-0502&C2DB=PRD&ResultTemplate=CII%2FCII
