# Supplementary material for: Association between the 2008–09 Seasonal Influenza Vaccine and Pandemic H1N1 Illness during Spring–Summer 2009: Four Observational Studies from Canada
Source: PLoS Med. 2010 Apr 6;7(4):e1000258. doi: 10.1371/journal.pmed.1000258 (PMC2850386; doi:10.1371/journal.pmed.1000258)
Supplement: Text S4 — Appendix D: Quebec population case-control study - Additional details. (0.05 MB PDF) [file pmed.1000258.s004.pdf]

Appendix D

Quebec population case-control study –
Additional details

Appendix D1. Quebec pH1N1 population case-control study – exclusion criteria and participation by study group

	Community pH1N1 cases	Hospitalized pH1N1 cases	Community controls
Reported cases / random phone numbers generated	993	415	2820
Phone number unknown	343	48	724*
No response/not reached	243	25	1082
Phone contact made	407	342	1014
Refusal	11	4	325
Not able to speak English/French	1	1	59
Before exclusions	395	337	630
Exclusions	11	67	27
< 6 month (Dec 1st 2008)	6	19	26
Hospitalized for reason other than pH1N1	N/A	16	N/A
Unknown vaccination status	5	33	1
Final participation	384	270	603

pH1N1= pandemic influenza A (H1N1); N/A=Not applicable

* Phone numbers generated by random digit dialling that were either invalid, not in service or for business

Appendix D2: Quebec pH1N1 population case-control study: participant profile

	Community pH1N1 cases (N=384)						Hospitalized pH1N1 cases (N=270)						Community controls (N=603)					
	6-59 mos	5-19 years	20-34 years	35-49 years	50-59 years	≥ 60 years	6-59 mos	5-19 years	20-34 years	35-49 years	50-59 years	≥ 60 years	6-59 mos	5-19 years	20-34 years	35-49 years	50-59 years	≥ 60 years
**N**	**12**	**158**	**89**	**62**	**44**	**19**	**35**	**73**	**40**	**50**	**37**	**35**	**40**	**201**	**101**	**114**	**88**	**59**
	n (%)	n (%)	n (%)	n (%)	n (%)	n (%)	n (%)	n (%)	n (%)	n (%)	n (%)	n (%)	n (%)	n (%)	n (%)	n (%)	n (%)	n (%)
**Symptoms**																		
Fever + cough	7 (58)	112 (71)	63 (72)	46 (74)	32 (73)	10 (52)	29 (85)	59 (81)	32 (80)	42 (84)	31 (84)	25 (71)	2 (5)	8 (4)	5 (5)	2 (2)	1 (1)	0
ILI	3 (25)	109 (69)	62 (70)	46 (74)	31 (70)	10 (53)	15 (43)	42 (57)	26 (65)	32 (64)	27 (73)	17 (48)	0	8 (4)	4 (4)	2 (2)	1 (1)	0
**Female**	5 (41)	75 (48)	56 (63)	36 (58)	30 (68)	14 (74)	15 (44)	26 (36)	27 (67)	31 (62)	25 (67)	20 (57)	20 (50)	101 (50)	67 (66)	74 (65)	50 (57)	41 (69)
**Chronic conditions**	3 (25)	42 (27)	15 (17)	11 (18)	12 (27)	13 (68)	14 (41)	37 (51)	28 (70)	30 (60)	24 (65)	30 (86)	0	13 (6)	17 (17)	19 (17)	20 (23)	26 (44)
**Smoker**†‡	N/A	5 (3)	16 (18)	12 (19)	9 (20)	1 (5)	N/A	2 (3)	11 (27)	14 (28)	11 (30)	9 (26)	N/A	7 (3)	21 (21)	23 (20)	26 (29)	8 (14)
**HCW**§	N/A	N/A	25 (28)	21 (34)	18 (41)	3 (16)	N/A	N/A	2 (5)	3 (6)	2 (5)	1 (3)	N/A	N/A	7 (7)	16 (14)	13 (15)	0
**TIV Status**																		
2008-09	5 (42)	49 (31)	32 (36)	24 (39)	26 (59)	9 (47)	16 (46)	24 (33)	11 (27)	24 (48)	15 (40)	29 (83)	10 (25)	18 (9)	16 (16)	26 (23)	19 (21)	30 (51)
2007-08‡	2 (40)	51 (32)	30 (34)	32 (52)	28 (64)	8 (42)	11 (55)	29 (39)	13 (32)	24 (49)	16 (43)	27 (82)	7 (29)	29 (14)	18 (17)	30 (26)	18 (20)	30 (51)
2007-08‡ only	0	12 (7)	5 (6)	9 (15)	3 (7)	0	3 (15)	6 (8)	5 (13)	3 (6)	2 (5)	0	5 (21)	13 (6)	6 (6)	9 (8)	0	1 (2)
2008-09 only	3 (25)	10 (6)	7 (8)	1 (2)	1 (2)	1 (5)	6 (17)	2 (3)	4 (10)	2 (4)	2 (5)	2 (6)	8 (20)	1 (0.5)	4 (4)	5 (4)	1 (1)	1 (2)
2007 & 2008‡	2 (40)	39 (25)	25 (28)	23 (38)	25 (57)	8 (42)	8 (40)	22 (30)	7 (17)	21 (43)	14 (38)	27 (82)	2 (8)	16 (8)	12 (11)	21 (18)	18 (20)	29 (49)
5 in 5 yrs	-	23 (15)	13 (15)	16 (26)	21 (48)	7 (37)	-	18 (25)	6 (15)	15 (30)	12 (30)	23 (66)	-	9 (5)	7 (7)	16 (14)	15 (17)	24 (41)
≥1 in 5 yrs	5 (42)	69 (44)	41 (46)	42 (68)	32 (73)	9 (47)	21 (60)	38 (52)	19 (47)	29 (58)	18 (48)	29 (83)	18 (45)	34 (17)	28 (28)	41 (36)	26 (29)	35 (59)
None in 5 yrs	-	86 (54)	48 (54)	20 (32)	12 (27)	9 (47)	-	22 (30)	20 (50)	19 (38)	16 (43)	4 (11)	-	167 (83)	73 (72)	73 (64)	62 (70)	24 (41)
1-3 in 5 yrs	-	34 (21)	22 (25)	21 (34)	9 (20)	2 (10)	-	13 (18)	9 (22)	11 (22)	6 (16)	0	-	16 (8)	20 (20)	20 (17)	9 (10)	10 (17)
4-5 in 5 yrs	-	31 (20)	19 (21)	21 (34)	23 (52)	7 (37)	-	21 (29)	8 (20)	16 (32)	13 (35)	26 (72)	-	11 (5)	9 (9)	23 (20)	17 (19)	25 (42)
**No chronic condition (N)**	9	116	74	51	32	6	20	36	12	20	13	5	40	188	84	95	68	33
TIV 2008-09	3 (33)	29 (25)	27 (37)	20 (39)	17 (53)	3 (50)	8 (40)	6 (17)	2 (17)	5 (25)	3 (23)	3 (60)	10 (25)	17 (9)	13 (15)	19 (20)	11 (16)	13 (40)
**With chronic conditions (N)**	3	42	15	11	12	13	15	37	28	30	24	30	0	13	17	19	20	26
TIV 2008-09	2 (67)	20 (48)	5 (33)	4 (36)	9 (75)	6 (46)	8 (53)	18 (49)	9 (32)	19 (63)	12 (50)	26 (87)	0	1 (8)	3 (18)	7 (34)	8 (40)	17 (65)

pH1N1= pandemic influenza A (H1N1); TIV=trivalent inactivated influenza vaccine; N/A=Not applicable
ILI=influenza-like illness: fever plus cough plus one or more of sore throat, myalgia, arthralgia, headache or prostration
†Smoker defined as: any current smoking
‡ For these variables denominators are slightly different due to missing or not applicable values
§HCW=Health care worker defined as: any person working in the health care system with or without direct contact with patients
NOTE: where percentages do not sum 100%, this is owing to missing data
