# Supplementary material for: Association between the 2008–09 Seasonal Influenza Vaccine and Pandemic H1N1 Illness during Spring–Summer 2009: Four Observational Studies from Canada
Source: PLoS Med. 2010 Apr 6;7(4):e1000258. doi: 10.1371/journal.pmed.1000258 (PMC2850386; doi:10.1371/journal.pmed.1000258)
Supplement: Text S5 — Appendix E: Ontario test-negative case-control study - Additional details. (0.05 MB PDF) [file pmed.1000258.s005.pdf]

Appendix E

Ontario test-negative case-control study –
Additional details

Appendix E1. Ontario pH1N1 test-negative case-control study: exclusion criteria and participation by study group

	Community pH1N1 cases	Hospitalized pH1N1 cases	pH1N1 test-negative community controls
Eligible			
Participating before exclusions	294	168	484
Exclusions			
Hospitalized among test-negatives			40
Missing vaccine status	15	5	10
Recent travel to Mexico	7	1	59
Without flu-like symptoms when tested	17	23	76
< 6 months of age	5	3	11
Final participation	250	136	288

pH1N1= pandemic influenza A (H1N1)

Appendix E2: Ontario pH1N1 test-negative case-control study: participant profile

	Community pH1N1 cases (N= 250)						Hospitalized pH1N1 cases (N= 136)						pH1N1 test-negative community controls (N= 288)					
	6-59 mos	5-19 years	20-34 years	35-49 years	50-59 years	≥ 60 years	6-59 mos	5-19 years	20-34 years	35-49 years	50-59 years	≥ 60 years	6-59 mos	5-19 years	20-34 years	35-49 years	50-59 years	≥ 60 years
**N**	**17** N(%)	**134** N(%)	**30** N(%)	**36** N(%)	**24** N(%)	**9** N(%)	**20** N(%)	**57** N(%)	**26** N(%)	**17** N(%)	**8** N(%)	**8** N(%)	**27** N(%)	**69** N(%)	**57** N(%)	**66** N(%)	**39** N(%)	**30** N(%)
**Symptoms (among "flu-like" participants)**																		
Fever + cough	11 (65)	98 (73)	17 (57)	23 (64)	16 (67)	2 (22)	14 (70)	44 (77)	15 (58)	12 (71)	6 (75)	6 (75)	11 (41)	32 (46)	21 (37)	26 (39)	11 (28)	7 (23)
ILI *	11 (65)	97 (72)	17 (57)	23 (64)	16 (67)	2 (22)	13 (65)	43 (75)	14 (54)	11 (65)	6 (75)	6 (75)	10 (37)	31 (45)	21 (37)	26 (39)	11 (28)	7 (23)
**Female**	12 (71)	87 (65)	17 (57)	19 (53)	16 (67)	4 (44)	14 (70)	42 (74)	16 (62)	13 (76)	4 (50)	5 (63)	20 (74)	50 (72)	37 (65)	38 (58)	26 (67)	17 (57)
**Chronic conditions**	1 (6)	16 (12)	1 (3)	1 (3)	11 (46)	5 (56)	3 (15)	24 (42)	6 (23)	7 (41)	7 (88)	4 (50)	2 (7)	11 (16)	6 (11)	8 (12)	8 (21)	11 (37)
**Smoker**†	N/A	5 (4)	8 (27)	4 (11)	5 (21)	0 (0)	N/A	1 (2)	9 (35)	4 (24)	4 (50)	2 (25)	N/A	6 (9)	25 (44)	17 (26)	10 (26)	4 (13)
**HCW**‡	N/A	N/A	5 (17)	5 (14)	5 (21)	2 (22)	N/A	N/A	6 (23)	1 (6)	1 (13)	0 (0)	N/A	N/A	8 (14)	9 (14)	2 (5)	3 (10)
**Median MD visits 12mos**	6.5	3	2	4	3	4	5.5	3	3	5	4	4	4	3	3	3	3	5
**TIV Status**																		
2008-09	7 (41)	56 (42)	9 (30)	17 (47)	13 (54)	7 (78)	4 (20)	20 (35)	7 (27)	4 (24)	5 (63)	6 (75)	6 (22)	15 (22)	16 (28)	22 (33)	17 (44)	20 (67)
2007-08	5 (29)	66 (49)	12 (40)	19 (53)	15 (63)	7 (78)	4 (20)	32 (56)	9 (35)	6 (35)	6 (75)	7 (88)	7 (26)	25 (36)	16 (28)	24 (36)	20 (51)	21 (70)
2007-08 only	0 (0)	17 (13)	6 (20)	3 (8)	2 (8)	0 (0)	2 (10)	12 (21)	2 (8)	3 (18)	2 (25)	1 (13)	2 (7)	13 (19)	7 (12)	7 (11)	4 (10)	2 (7)
2008-09 only	2 (12)	7 (5)	3 (10)	1 (3)	0 (0)	0 (0)	2 (10)	0 (0)	0 (0)	0 (0)	1 (6)	1 (13)	1 (4)	3 (4)	7 (12)	5 (8)	1 (3)	1 (3)
2007 & 2008	5 (29)	49 (37)	6 (20)	16 (44)	13 (54)	7 (78)	2 (10)	20 (35)	7 (27)	3 (18)	4 (50)	6 (75)	5 (19)	12 (17)	9 (16)	17 (26)	16 (41)	19 (63)
5 in 5 years	N/A	38 (28)	5 (17)	11 (31)	9 (38)	6 (67)	N/A	16 (28)	6 (23)	3 (18)	4 (50)	5 (63)	N/A	9 (13)	5 (9)	13 (20)	13 (33)	16 (53)
≥1 in 5 yrs	7 (41)	90 (67)	17 (57)	25 (69)	17 (71)	8 (89)	7 (35)	45 (79)	14 (54)	8 (47)	7 (88)	7 (88)	8 (30)	36 (52)	30 (53)	38 (58)	24 (62)	25 (83)
None in 5 yrs	10 (59)	44 (33)	13 (43)	11 (31)	7 (29)	1 (11)	13 (65)	12 (21)	12 (46)	9 (53)	1 (13)	1 (13)	19 (70)	33 (48)	27 (47)	28 (42)	15 (38)	5 (17)
1-3 in 5 yrs	4 (24)	38 (28)	12 (40)	10 (28)	8 (33)	0 (0)	7 (35)	20 (35)	8 (31)	4 (24)	2 (25)	1 (13)	6 (22)	22 (32)	20 (35)	21 (32)	10 (26)	9 (30)
4-5 in 5 yrs	3 (18)	52 (39)	5 (17)	15 (42)	9 (38)	8 (89)	12 (21)	25 (44)	6 (23)	4 (24)	5 (63)	6 (75)	2 (7)	14 (20)	10 (18)	17 (26)	14 (36)	16 (53)
**No chronic condition (N)**	16	115	29	31	12	4	17	32	19	10	1	4	24	57	49	55	29	16
TIV 2008-09	7 (44)	47 (41)	9 (31)	17 (55)	7 (58)	3 (75)	3 (18)	12 (38)	5 (26)	1 (10)	0 (0)	2 (50)	5 (21)	9 (16)	13 (27)	20 (36)	12 (41)	9 (56)
**With chronic conditions (N)**	1	16	1	1	11	5	3	24	6	7	7	4	2	11	6	8	8	11
TIV 2008-09	0 (0)	6 (38)	0 (0)	0 (0)	6 (55)	4 (80)	1 (33)	8 (33)	2 (33)	3 (43)	5 (71)	4 (100)	1 (50)	6 (55)	1 (17)	2 (25)	4 (50)	8 (73)

pH1N1= pandemic influenza A (H1N1); TIV=trivalent inactivated influenza vaccine; *ILI=influenza-like illness: fever plus cough plus one or more of sore throat, myalgia, arthralgia, headache or prostration; N/A=Not applicable; † Smoker defined as: Ever smoked a cigarette in the past 6 months; ‡HCW=Health care worker defined as: doctor, nurse or other health care professional.
NOTE: where percentages do not sum 100%, this is owing to missing data
