# Supplementary material for: Association between the 2008–09 Seasonal Influenza Vaccine and Pandemic H1N1 Illness during Spring–Summer 2009: Four Observational Studies from Canada
Source: PLoS Med. 2010 Apr 6;7(4):e1000258. doi: 10.1371/journal.pmed.1000258 (PMC2850386; doi:10.1371/journal.pmed.1000258)
Supplement: Text S6 — Appendix F: Overall and antigenic region identity of 2009 pandemic H1N1 (pH1N1) proteins compared to representative recently circulating and vaccine strains of A/H1N1 and A/H3N2. (0.03 MB PDF) [file pmed.1000258.s006.pdf]

Appendix F

Overall and antigenic region identity of 2009 pandemic H1N1 (pH1N1) proteins compared to representative recently circulating and vaccine strains of A/H1N1 and A/H3N2

Appendix F. Overall and antigenic region identity of 2009 pandemic H1N1 (pH1N1) proteins compared to representative recently circulating and vaccine strains of A/H1N1 and A/H3N2

Methods:

To quantify the overall identity between the proteins of pH1N1 and those of recently circulating (1974-2008) seasonal A/H1N1 and A/H3N2 viruses, we performed a BLAST [1] search of each of the ten pH1N1 (A/California/04/2009(H1N1)) proteins against a database of 249 proteins from representative A/H1N1 and A/H3N2 virus isolates (restricted to H1N1 viruses for the HA and NA surface proteins). The 36 representative isolates[1] were selected based upon their inclusion in WHO vaccine recommendations from 1974 onward and availability of protein sequence in the NCBI Influenza Virus Resource.

We also performed a more focused comparison, measuring the similarity at the level of antigenic regions. Antigenic regions for hemagglutinin (HA) and neuraminidase (NA) were reported in Soundararajan et al [2], while antigenic regions for the other proteins were determined through mapping of influenza epitopes from the Immune Epitope Database (IEDB, http://www.immuneepitope.org, [3]). IEDB was queried for all influenza A epitopes, with the results filtered to remove: i) epitopes in viruses other than H1N1 and H3N2, ii) epitopes from non-human viruses or other organisms, iii) single-residue epitopes, iv) epitopes that could not be assigned to a protein due to incomplete annotation, v) epitopes from prior to 1977, and vi) epitopes from HA and NA.

Aligning epitope sequences to each protein allowed the putative antigenic regions to be extracted. For two proteins, NP and M1, the mapped epitopes covered the entirety of the protein. In lieu of these epitopes, alternative annotations were used to identify relevant antigenic regions of NP and M1. Literature searches revealed the following common epitopes: NP35-42 [4], NP217–225 [5], NP366–374[6], M1-17-31 & M1-58-66 [7], M1-128-135 [4]. The antigenic regions of each A/California/04/2009 protein were concatenated and BLASTed against the concatenated antigenic regions of that protein's respective best recent BLAST hit to quantify identity at the antigenic level. For each protein, the best and worst overall (1974-2008) and recent (2000-2008) hits are indicated in Table F1. Only sequences of matching length were considered.

Results:

There was little dissimilarity in the internal polymerase proteins (95-98% identity both overall and in antigenic regions), regardless of timeframe (1974-2008 or 2000-2008) or subtype (Table F1). The M1 protein was also well-conserved between pH1N1 and recent human H1N1 isolates (94%), particularly with respect to its antigenic regions (97%), while the internal nucleoprotein (NP) displayed 85-92% homology with recent H3N2 strains. Taken together, there is marked degree of conservation amongst key internal proteins of pH1N1 and seasonal influenza (notably M1, NP and polymerases) for both recent (2000 onwards) and more historical (1974-2000) viruses. Conversely, the HA and NA surface components of the virus were quite divergent from human H1N1 viruses. It is notable that while the best hits to pH1N1 HA and NA exhibited 81% (1986 H1) and 83% (1978 N1) overall identity, respectively, this dropped to 65% and 37% when their antigenic regions were considered.

References

1. Altschul SF, Gish W, Miller W, Myers EW, Lipman DJ. (1990) Basic local alignment search tool. J Mol Biol 215;403-410.
2. Soundararajan V, Tharakaraman K, Raman R, Raguram S, Shriver Z, Sasisekharan V, Sasisekharan R. (2009) Extrapolating from sequence--the 2009 H1N1 'swine' influenza virus. Nat Biotechnol. 27(6):510-3.
3. Peters B, Sidney J, Bourne P, Bui HH, Buus S, Doh G, Fleri W, Kronenberg M, Kubo R, Lund O, Nemazee D, Ponomarenko JV, Sathiamurthy M, S choenberger S, Stewart S, Surko P, Way S, Wilson S, Sette A. (2005). The immune epitope database and analysis resource: from vision to blueprint. PLoS Biol. 3(3):e91
4. Zhong W, Reche PA, Lai CC, Reinhold B, Reinherz EL. (2003) Genome-wide characterization of a viral cytotoxic T lymphocyte epitope repertoire. J Biol Chem. 278(46):45135-44.
5. Thomas PG, Brown SA, Keating R, Yue W, Morris MY, So J, Webby RJ, Doherty PC. (2007). Hidden epitopes emerge in secondary influenza virus-specific CD8+ T cell responses. J Immunol. 178(5):3091-8.
6. Schild H, Norda M, Deres K, Falk K, Rötzschke O, Wiesmüller KH, Jung G, Rammensee HG. (1991) Fine specificity of cytotoxic T lymphocytes primed in vivo either with virus or synthetic lipopeptide vaccine or primed in vitro with peptide. J Exp Med. 174(6):1665-8.
7. Jameson J, Cruz J, Terajima M, Ennis FA. (1999) Human CD8+ and CD4+ T lymphocyte memory to influenza A viruses of swine and avian species. J Immunol. 162(12):7578-83.

[1] A/Bangkok/01/1979 (H3N2), A/Bayern/7/95 (H1N1), A/Beijing/262/95 (H1N1), A/Beijing/32/92 (H3N2), A/Beijing/353/89 (H3N2), A/Brazil/11/78 (H1N1), A/Brisbane/10/2007 (H3N2), A/Brisbane/59/2007 (H1N1), A/California/7/2004 (H3N2), A/Chile/1/83 (H1N1), A/Fujian/411/2002 (H3N2), A/Hiroshima/52/2005 (H3N2), A/Johannesburg/33/94 (H3N2), A/Leningrad/360/1986 (H3N2), A/Moscow/10/99 (H3N2), A/Nanchang/933/95 (H3N2), A/New Caledonia/20/1999 (H1N1), A/New York/55/2004 (H3N2), A/Panama/2007/1999 (H3N2), A/Philippines/2/82 (H3N2), A/Port Chalmers/1/1973 (H3N2), A/Shangdong/9/93 (H3N2), A/Shanghai/11/87 (H3N2), A/Sichuan/02/87 (H3N2), A/Singapore/6/1986 (H1N1), A/Solomon Islands/3/2006 (H1N1), A/South Dakota/6/2007 (H1N1), A/Sydney/5/97 (H3N2), A/Texas/36/91 (H1N1), A/Uruguay/716/2007 (H3N2), A/USSR/90/77 (H1N1), A/Victoria/3/75 (H3N2), A/Wellington/1/2004 (H3N2), A/Wisconsin/67/2005 (H3N2), A/Wuhan/359/95 (H3N2), A/Wyoming/03/2003 (H3N2).

Table F1. pH1N1 overall and antigenic identity to recently circulating influenza viruses.

		IDENTITY	
		Overall	Antigenic Regions
**PB2**			
Best Hit 1974-2008	A/USSR/90/1977*	95.12%	94.82%
Best Recent Hit 2000-2008	A/NewYork/55/2004**	94.73%	92.83%
Worst Hit 1974-2008	A/Beijing/262/1995*	93.93%	92.83%
Worst Recent Hit 2000-2008	A/Brisbane/10/2007**	94.46%	92.83%
**PB1**			
Best Hit 1974-2008	A/Shangdong/9/1993**	98.15%	98.22%
Best Recent Hit 2000-2008	A/Wellington/01/2004**	97.62%	97.55%
Worst Hit 1974-2008	A/Chile/1/1983*	94.31%	94.88%
Worst Recent Hit 2000-2008	A/Wisconsin/67/2005**	97.09%	97.10%
**PA**			
Best Hit 1974-2008	A/Chile/1/1983*	95.25%	95.86%
Best Recent Hit 2000-2008	A/Wisconsin/67/2005**	94.00%	94.14%
Worst Hit 1974-2008	A/Hiroshima/52/2005**	93.58%	93.79%
Worst Recent Hit 2000-2008	A/Hiroshima/52/2005**	93.58%	93.79%
**HA[a]**			
Best Hit 1974-2008	A/Singapore/6/1986*	80.74%	64.79%
Best Recent Hit 2000-2008	A/SolomonIslands/3/2006*	79.68%	57.14%
Worst Hit 1974-2008	A/SouthDakota/06/2007*	78.80%	55.71%
Worst Recent Hit 2000-2008	A/SouthDakota/06/2007*	78.80%	55.71%
**NP**			
Best Hit 1974-2008	A/Sydney/5/1997**	91.57%	92.31%
Best Recent Hit 2000-2008	A/Wisconsin/67/2005**	90.36%	84.62%
Worst Hit 1974-2008	A/Beijing/262/1995*	89.16%	80.77%
Worst Recent Hit 2000-2008	A/NewYork/55/2004**	89.56%	84.62%
**NA[a]**			
Best Hit 1974-2008	A/Brazil/11/1978*	82.98%	37.25%
Best Recent Hit 2000-2008	A/SouthDakota/06/2007*	81.28%	35.29%
Worst Hit 1974-2008	A/Bayern/7/1995*	81.06%	33.33%
Worst Recent Hit 2000-2008	A/Brisbane/59/2007*	81.16%	35.29%
**M1**			
Best Hit 1974-2008	A/USSR/90/1977*	94.44%	96.77%
Best Recent Hit 2000-2008	A/SolomonIslands/3/2006*	93.65%	96.77%
Worst Hit 1974-2008	A/NewYork/55/2004**	91.27%	96.77%
Worst Recent Hit 2000-2008	A/NewYork/55/2004**	91.27%	96.77%
**M2**			
Best Hit 1974-2008	A/Brazil/11/1978*	85.57%	86.67%
Best Recent Hit 2000-2008	A/Hiroshima/52/2005**	76.19%	85.33%
Worst Hit 1974-2008	A/SolomonIslands/3/2006*	73.81%	82.67%
Worst Recent Hit 2000-2008	A/SolomonIslands/3/2006*	73.81%	82.67%
**NS1**			
Best Hit 1974-2008	A/Chile/1/1983*	83.11%	83.00%
Best Recent Hit 2000-2008	A/NewYork/55/2004**	78.54%	78.00%
Worst Hit 1974-2008	A/Leningrad/360/1986**	77.63%	78.00%
Worst Recent Hit 2000-2008	A/Wyoming/03/2003**	78.08%	77.00%
**NS2**			
Best Hit 1974-2008	A/Brisbane/10/2007**	90.08%	86.96%
Best Recent Hit 2000-2008	A/Wellington/01/2004**	90.08%	89.13%
Worst Hit 1974-2008	A/Beijing/262/1995*	87.60%	89.13%
Worst Recent Hit 2000-2008	A/Fujian/411/2002**	89.26%	89.13%

pH1N1= pandemic influenza A (H1N1)
*A/H1N1; **A/H3N2
[a]Analysis restricted to A/H1N1 viruses for the HA and NA surface proteins.
NOTE: A/SouthDakota/06/2007 and A/Brisbane/59/2007 considered antigenically equivalent as 2008-09 H1N1 vaccine components based on HA. Similarly, A/Hiroshima/52/2005 and A/Wisconsin/67/2005 considered antigenically equivalent as 2006-07 H3N2 vaccine components based on HA.
