# Supplementary material for: Association between the 2008–09 Seasonal Influenza Vaccine and Pandemic H1N1 Illness during Spring–Summer 2009: Four Observational Studies from Canada
Source: PLoS Med. 2010 Apr 6;7(4):e1000258. doi: 10.1371/journal.pmed.1000258 (PMC2850386; doi:10.1371/journal.pmed.1000258)
Supplement: Text S7 — Appendix G: Theoretical analysis of whether a 2-fold increased risk of pH1N1 associated with prior seasonal influenza vaccination could be explained by TIV effectively blocking the heterosubtypic cross-immunity provided by prior seasonal influenza infection. (0.06 MB PDF) [file pmed.1000258.s007.pdf]

Appendix G

Theoretical analysis of whether a 2-fold increased risk of pH1N1 associated with prior seasonal influenza vaccination could be explained by trivalent inactivated influenza vaccine (TIV) effectively blocking the heterosubtypic cross-immunity provided by prior seasonal influenza infection

Appendix G. Can a two-fold increased risk of pandemic influenza A/H1N1 (pH1N1) associated with prior seasonal influenza vaccination be explained by vaccine effectively blocking the heterosubtypic cross-immunity provided by seasonal influenza infection?

We have preliminarily explored this hypothesis on theoretical grounds. Based on the schematic below for pH1N1 in TIV vaccinated and unvaccinated groups taking into account prior seasonal influenza and assuming:

a) 100 people who received seasonal trivalent inactivated influenza vaccine (TIV VACCINATED)
b) 100 people who did not receive seasonal TIV [NOT TIV VACCINATED]
c) A seasonal influenza infection attack rate (AR) of 70% (single season or accumulated across seasons)
   - C=70;
   - A=100-70=30
d) A TIV vaccine effectiveness (VE) of 50% vs. (pH1N1 immunity-inducing) seasonal influenza infection
   - b=VE*C=0.5*70 = 35;
   - c=(1-VE)*C=(1 - 0.5)*70 = 35;
   - a=100 - (b+c) = 100 – (35 +35) =30
e) A 10% pH1N1 attack rate (AR) during the spring/summer 2009 in Canada
   - θ_1=0.10

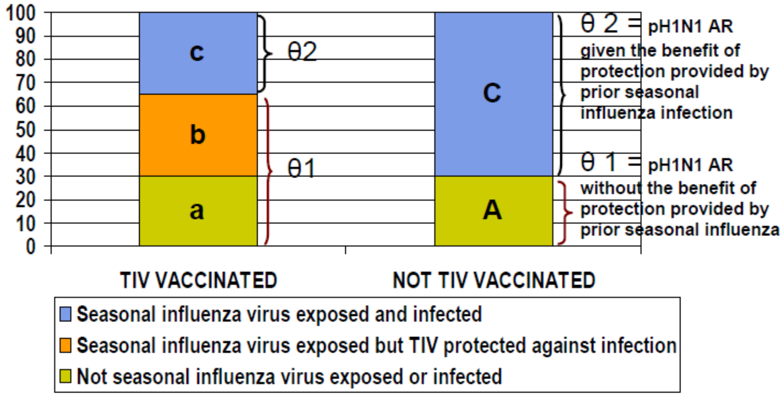

Then we can derive the required attack rate for pH1N1 infection (θ2) to yield a risk ratio for pH1N1 infection of 2.0 for the vaccinated compared to the unvaccinated, based on the equation below for RR:

$$RR_{pH1N1} Vacc / Unvacc = \frac{[(a+b)*\theta 1 + (c*\theta 2)]}{(A*\theta 1)+(C*\theta 2)}$$

Thus, 2 = [(30+35)*0.1 + (35* θ2)] / [30*0.1] + (70* θ2)]
θ2=0.5/105 = 0.005

This means that the pH1N1 attack rate would be reduced from 10% (θ_1=0.10) in those without the benefit of prior seasonal infection to 0.5% (θ_2=0.005) in those who had the postulated benefit of prior seasonal influenza infection – in other words, it would require that seasonal influenza infection provides cross-protection of >95% against pH1N1 [(θ_1- θ_2)/ θ_1], which seems implausible given that the pandemic with assumed 10% attack rate occurred in that same immuno-epidemiologic context.

We can vary these assumptions, noting that plausibility is driven by prior seasonal influenza AR, seasonal influenza infection-induced cross-immunity to pH1N1, or TIV block of that and that the RR is unaffected by θ_1.

If we assume a higher seasonal influenza attack rate (95%) over several seasons and repeat annual vaccination with effectiveness in blocking that of 50% (C=95; A=5; a=5; b=47.5; c=47.5; θ_1=0.10), then to achieve a relative risk of 2.0 would require θ2=0.0298 – in other words, a reduction in the pH1N1 AR from 10% in those without the benefit of seasonal infection to 3% in those who had benefited from prior seasonal infection. It may be debatable whether 70% cross-protection against pH1N1 [(θ_1- θ_2)/ θ_1] could be afforded by seasonal influenza infection but this again seems unlikely.

With assumptions of seasonal influenza attack rates below 50%, the hypothesis becomes completely unsupported (RR falls below 2) even assuming seasonal influenza infection induces 100% cross-protection against pH1N1 (θ2=0), unless TIV protection (VE) against that seasonal influenza infection is also assumed to be 100%.
